# The effects of SGLT2 inhibitors on atrial fibrillation recurrence and cardiac function after catheter ablation in patients with atrial fibrillation and heart failure: a retrospective cohort study and meta-analysis

**DOI:** 10.3389/fcvm.2026.1827339

**Published:** 2026-05-01

**Authors:** Xueying Gao, Zhanxin Zhang, Kexin Chen, Yaqiong Jin, Fei Chen, Jie Zhang, Lu Geng, Keke Wang, Cunliang Gao, Li Wang, Jingchao Lu

**Affiliations:** Department of Cardiology, The Second Hospital of Hebei Medical University, Shijiazhuang, Hebei Province, China

**Keywords:** atrial arrhythmia recurrence, atrial fibrillation, cardiac function, heart failure, SGLT2 inhibitors

## Abstract

**Objective:**

The impact of Sodium-glucose cotransporter 2 inhibitors (SGLT2i) on postoperative atrial fibrillation (AF) recurrence and cardiac function in patients with AF and heart failure (HF) undergoing catheter ablation remains unclear.

**Methods and results:**

This study comprised two parts. Part 1 was a retrospective cohort study involving 168 patients with AF and HF who underwent first-time catheter ablation (SGLT2i group: *n* = 83; non-SGLT2i group: *n* = 85). Atrial arrhythmia recurrence rate was significantly lower in the SGLT2i group compared to the non-SGLT2i group (13.25% vs. 25.88%, *P* = 0.039). Multivariate Cox proportional hazards regression analysis identified severe atrial fibrosis (HR 2.80, 95% CI: 1.13–6.96, *P* = 0.026) and SGLT2i use (HR 0.45, 95% CI: 0.22–0.94, *P* = 0.033) as independent predictors of atrial arrhythmia recurrence. At 12-month follow-up, SGLT2i group had a smaller left atrial diameter (LA: 37.37 ± 4.25 mm vs. 39.25 ± 6.22 mm, *P* = 0.024) and a higher left ventricular ejection fraction (LVEF: 62.06 ± 11.39% vs. 58.89 ± 7.98%, *P* = 0.039). Part 2 was a meta-analysis. We systematically searched and included 13 studies-2 RCTs and 11 retrospective cohort studie, involving 7,954 patients (SGLT2i: 3518; non-SGLT2i: 4436). Results indicated that SGLT2i significantly reduced the risk of atrial arrhythmia recurrence after catheter ablation (RR = 0.59, 95% CI: 0.51–0.69, *P* < 0.001). Furthermore, SGLT2i was associated with reduced risks of all-cause mortality (RR: 0.66, 95% CI: 0.48–0.91, *P* = 0.011).

**Conclusion:**

In patients with AF and HF, SGLT2i use is associated with a lower risk of AF recurrence after catheter ablation and with improvements in cardiac structure and function. SGLT2i is also associated with lower risks of all-cause mortality, rehospitalization, and HF events.

## 1 Introduction

Atrial fibrillation (AF) can induce heart failure (HF) or exacerbate pre-existing HF. Studies show that up to 30% of patients present with both conditions. HF patients often have a poor prognosis, with a 5-year survival rate of less than 50% (1). For patients with coexisting HF and AF, achieving long-term sinus rhythm maintenance and improving cardiac function remain critical and challenging clinical goals. Catheter ablation (CA), as a first-line treatment for AF, has been widely adopted in clinical practice (2). Research indicates that compared to patients without HF, those with AF and HF have a significantly higher AF recurrence rate post-ablation. Therefore, comprehensive post-ablation management is crucial for maintaining sinus rhythm. Multiple studies have shown that SGLT2 inhibitors (SGLT2i) can effectively improve cardiac function and reduce HF hospitalization and mortality risks. SGLT2i has become an integral part of guideline-directed medical therapy for HF, and current guidelines recommend it as a Class IA first-line therapy for patients across the full ejection fraction spectrum (3). Recent studies suggest that SGLT2i may also have a beneficial effect in delaying the onset and progression of AF, making it a current research hotspot in the AF treatment field.

Research indicates that SGLT2i may reduce AF occurrence by mechanisms such as modulating intracellular Na⁺ and Ca²⁺ disturbances, improving mitochondrial function, reducing atrial myocardial fat infiltration, and suppressing inflammatory responses, thereby reversing cardiac structural and electrical remodeling (4). Studies in diabetic rat models have shown that empagliflozin significantly reduces AF incidence and inhibits left atrial enlargement and fibrosis (5). Lin et al. administered standard therapeutic doses of dapagliflozin to HF rats for six consecutive weeks. The results showed that dapagliflozin-treated rats exhibited lower AF incidence and shorter AF duration, along with significant attenuation of myocardial fibrosis, reduced cardiomyocyte apoptosis, and decreased expression of endoplasmic reticulum stress-related proteins (6). Building on this theoretical foundation, clinical studies provide further corroboration. One study involving 399,810 patients diagnosed with type 2 diabetes showed that those taking SGLT2 inhibitors had a lower risk of arrhythmias compared to those not taking them (7). Another meta-analysis encompassing 22 clinical studies and 52,115 patients found that, compared to placebo, SGLT2i treatment reduced the risk of AF and atrial flutter by 18% and 17%, respectively (8).

However, the impact of SGLT2i on atrial arrhythmia recurrence outcomes and cardiac function in patients with AF and HF after catheter ablation remains unclear. This study consists of two components: 1. A retrospective cohort study analyzing data from AF patients with HF post-catheter ablation, comparing the SGLT2i and non-SGLT2i groups to investigate whether SGLT2i can reduce post-ablation AF recurrence and improve cardiac function in this population. 2. Building upon this, a meta-analysis incorporating original clinical studies on SGLT2i use after AF catheter ablation was conducted to systematically evaluate the impact of SGLT2i on post-ablation atrial arrhythmia recurrence and prognosis, providing more comprehensive and detailed evidence for advancements in maintaining sinus rhythm post-AF ablation.

## 2 Study Part 1: Cohort Study

### 2.1 Materials and methods

#### 2.1.1 Study population

This was a retrospective study. Consecutive patients diagnosed with atrial fibrillation and heart failure who underwent their first catheter ablation procedure at the Department of Cardiology, The Second Hospital of Hebei Medical University, between March 2022 and February 2024, were enrolled.

This study was approved by the Scientific Research Ethics Committee of The Second Hospital of Hebei Medical University (Ethical Review Approval Number: 2022-R531).

Inclusion criteria:

(1) Age ≥18 years.(2) Meeting guideline-recommended diagnostic criteria for non-valvular atrial fibrillation [Atrial fibrillation was diagnosed by a single-lead electrocardiogram (ECG) showing an episode lasting ≥30 s or a 12-lead ECG showing an episode lasting ≥10 s, characterized by the absence of *P* waves, replaced by fibrillatory waves (f waves) of variable size, morphology, and timing, with an absolutely irregular ventricular response. Paroxysmal atrial fibrillation was defined as AF terminating spontaneously or by intervention within 7 days. Persistent atrial fibrillation was defined as AF lasting ≥7 days). (3) Coexisting heart failure diagnosed according to the 2021 ESC Universal Definition of Heart Failure, requiring symptoms and/or signs of HF accompanied by objective evidence of cardiac dysfunction (e.g., elevated natriuretic peptides or structural/functional abnormalities on echocardiography). (4) First-time catheter ablation procedure.

Grouping:

Non-SGLT2i Group: Received conventional treatment including antiarrhythmic drugs, anticoagulants, etc.

SGLT2i Group: Received conventional treatment plus an SGLT2 inhibitor (10 mg once daily).

The decision to prescribe SGLT2 inhibitors was made by the attending cardiologist based on current heart failure guidelines (presence of HFrEF, HFmrEF, or HFpEF with additional metabolic indications), irrespective of diabetes status. The specific drugs are: dapagliflozin or empagliflozin 10 mg per day.

**Exclusion Criteria:** (1) Congenital heart disease or acute myocardial infarction patients. (2) Patients with severe hepatic or renal insufficiency: Alanine aminotransferase (ALT) > 120 U/L or Aspartate aminotransferase (AST) > 120 U/L, or estimated glomerular filtration rate (eGFR) <30 mL/min/1.73 m^2^. (3) Patients with malignancy. (4) Patients with valvular atrial fibrillation. (5) Patients with missing clinical or follow-up data.

### 2.2 Study methods

#### 2.2.1 Preoperative data collection

Basic information such as age and gender, as well as past medical history, were collected upon patient admission. Laboratory tests performed on the second day of hospitalization were recorded, including complete blood count, liver function tests, renal function tests, lipid profile, B-type natriuretic peptide (BNP), and inflammatory markers. Data from transthoracic echocardiography assessing cardiac chamber size, cardiac function, valvular lesions, and wall motion were documented. Transesophageal echocardiography was performed to rule out atrial and atrial appendage thrombi. Cardiac computed tomography was used to evaluate the morphology and structure of the left atrium and pulmonary veins. All patients underwent CHA₂DS₂-VASc and HAS-BLED risk scoring. Antiarrhythmic drugs were discontinued for at least five half-lives prior to the ablation procedure. Anticoagulant medications were withheld on the day of the procedure. The condition and procedural details were explained to the patients, and informed consent for the interventional examination and treatment was obtained.

#### 2.2.2 Procedural details

Patients were placed in the supine position. After connecting to the multi-channel electrophysiology recorder and the Carto3.0 three-dimensional mapping system, the skin was disinfected and sterile drapes were applied. Local anesthesia was achieved using lidocaine. The left femoral vein was punctured, and an 8F sheath was inserted. A steerable coronary sinus electrode catheter was advanced to the distal coronary sinus. The right femoral vein was punctured, an 8F sheath was placed, and a transseptal puncture was performed. After successful puncture, unfractionated heparin was administered intravenously at a dose of 100 IU/kg. Activated clotting time (ACT) was monitored hourly during the procedure and maintained at approximately 250–300 s. Using a high-density mapping catheter guided by the Carto3.0 three-dimensional mapping system, three-dimensional geometry reconstruction of the left atrium and pulmonary veins was performed, followed by substrate mapping. All patients underwent bilateral pulmonary vein isolation (PVI). For patients with persistent atrial fibrillation, after completing PVI, additional linear ablation lesions (such as left atrial roof line, posterior wall box, mitral isthmus, or tricuspid isthmus ablation) were performed in some patients based on the specific case. If sinus rhythm was not restored, electrical cardioversion was performed. The procedural endpoints were electrical isolation of the pulmonary veins and achievement of bidirectional conduction block in the linear ablation lines (where applicable). When sinus rhythm was restored during the procedure, electroanatomic voltage mapping (EAVM) was performed to assess the extent of atrial fibrosis. Fibrosis was categorized as: Low-grade fibrosis: FACM (Grade I + II); High-grade fibrosis: FACM (Grade III + IV) [15].

#### 2.2.3 Postoperative management

Patients underwent sandbag compression at the bilateral femoral puncture sites for 6 h for hemostasis. Subcutaneous low-molecular-weight heparin was initiated 4 h post-procedure. A postoperative ECG was obtained, and cardiac rhythm was continuously monitored. Patients were discharged 48–72 h after the procedure if their condition was stable. At discharge, patients with a heart rate >60 beats per minute received oral amiodarone or dronedarone (administered for 1 month for paroxysmal AF patients and for 3 months for persistent AF patients). If the heart rate decreased to <50 beats per minute or the QT interval prolonged to >0.48 s during medication use, amiodarone or dronedarone was discontinued. All patients received novel oral anticoagulants (NOACs) routinely for 3 months post-procedure. All patients received oral proton pump inhibitors for 1 month. Patients who experienced AF recurrence at 3 months post-procedure and had a CHA_2_DS_2_-VASc score greater than 2 points had their novel oral anticoagulant therapy re-initiated.

#### 2.2.4 Follow-up

Holter monitoring was performed at 1, 3, 6, and 12 months post-procedure during scheduled outpatient visits. Additional ECG recordings were obtained if patients reported palpitations, dizziness, or other symptoms suggestive of arrhythmia. No other implantable loop recorders were used. Follow-up data were collected during outpatient visits at 1, 3, 6, and 12 months post-procedure, including: 1. Basic information, such as results from complete blood count, coagulation profile, and comprehensive biochemistry panel. By routine electrocardiogram and 24-hour ambulatory holter monitoring, atrial arrhythmia recurrence events were recorded, defined as any episode of atrial fibrillation, atrial flutter, or atrial tachycardia lasting more than 30 s occurring after the 3-month blanking period following the radiofrequency ablation procedure. The timing of atrial arrhythmia recurrence was also recorded. 3. Echocardiographic parameters (including LA dimension, LV dimension, LVEF, E/e′ ratio, E/A ratio, etc., measured at each follow-up).

### 2.3 Statistical analysis

Analyses were performed using R software (version 4.4.0). Continuous variables conforming to a normal distribution are presented as mean ± standard deviation, and comparisons between the two groups were made using the *t*-test. Non-normally distributed data are presented as median (interquartile range), and comparisons between groups were made using the Mann–Whitney *U*-test (rank-sum test). Categorical variables are presented as numbers (percentages), and comparisons between groups were made using the Chi-square test. Repeated-measures analysis of variance (ANOVA) was used to compare cardiac ultrasound parameters between the two groups across different time points. Kaplan–Meier curves were plotted to compare the rates of freedom from atrial fibrillation recurrence between the two groups after ablation. Univariate and multivariate Cox proportional hazards regression analyses were performed to identify independent predictive factors associated with AF recurrence. Due to the retrospective design and limited sample size, propensity score matching was not feasible. However, multivariable Cox regression was performed to adjust for predefined confounders including age, sex, persistent AF, left atrial fibrosis grade, and left atrial diameter, et al. Subgroup analyses were conducted. A *P*-value < 0.05 was considered statistically significant.

## 3 Results for study part 1

### 3.1 Baseline characteristics

A total of 202 patients with atrial fibrillation and heart failure scheduled for first-time catheter ablation were consecutively enrolled. After excluding 18 patients with valvular atrial fibrillation, 1 patient with congenital heart disease, 2 patients with malignancy, and 13 patients with missing clinical or follow-up data, 168 patients were finally included. Based on whether SGLT2 inhibitors were used, patients were divided into two groups: the SGLT2i group (*n* = 83) and the non-SGLT2i group (*n* = 85). In the SGLT2i group, 56 patients received dapagliflozin (10 mg once daily) and 27 received empagliflozin (10 mg once daily). Due to the small sample size for each drug, separate comparative analyses were not performed.

In the SGLT2i group, the mean age was 63.73 ± 10.79 years, 68.67% were male, 65.06% had persistent atrial fibrillation, and 63.86% had heart failure with preserved ejection fraction (HFpEF). In the non-SGLT2i group, the mean age was 64.32 ± 8.99 years, 60.00% were male, 77.65% had persistent atrial fibrillation, and 71.76% had HFpEF. The proportion of patients with diabetes was 25.30% in the SGLT2i group and 18.82% in the non-SGLT2i group. Analysis of basic characteristics showed no significant differences between the two groups in terms of age, gender, CHA₂DS₂-VASc score, HAS-BLED score, BMI, comorbidities (hypertension, diabetes mellitus, hyperlipidemia, coronary artery disease, stroke), New York Heart Association (NYHA) functional class, heart failure type, or atrial fibrillation type (see Table 1 for details) (all *P* > 0.05). Analysis of relevant laboratory tests also showed no significant differences (see Table 2 for details) (*P* > 0.05). Analysis and comparison of procedure-related indicators between the two groups showed no significant differences in left atrial fibrosis degree (low-grade fibrosis, high-grade fibrosis) or ablation strategies (circumferential pulmonary vein isolation, left atrial roof line ablation, posterior wall box ablation, tricuspid isthmus ablation, mitral isthmus ablation) (*P* > 0.05) (see Table 3 for details). The use of medications post-procedure, including anticoagulants, antiarrhythmic drugs, beta-blockers, ACEI/ARB/ARNI drugs, and mineralocorticoid receptor antagonists, was largely similar between the two groups (*P* > 0.05) (see Table 4 for details).

**Table 1:** Comparison of preoperative general clinical data between the Two groups of patients.

Characteristic	SGLT2i group (*n* = 83)	Non-SGLT2i group (*n* = 85)
Age [years, Mean ± SD]	63.73 ± 10.79	64.32 ± 8.99
BMI[kg/m2, Mean ± SD]	26.78 ± 3.84	25.80 ± 3.02
HAS-BLED score [median (IQR)]	1.00 (1.00,2.00)	1.00 (1.00,2.00)
CHA2DS2-VASc score [median (IQR)]	3.00 (2.00,4.00)	3.00 (2.00,4.00)
Gender		
Male [n (%)]	57 (68.67)	51 (60.00)
Female [n (%)]	26 (31.33)	34 (40.00)
Smoking history [n (%)]	28 (33.73)	26 (30.59)
Alcohol consumption history [n (%)]	30 (36.14)	20 (23.53)
Comorbidities		
Hypertension [n (%)]	44 (53.01)	39 (45.88)
Diabetes [n (%)]	21 (25.30)	16 (18.82)
Coronary heart disease [n (%)]	12 (14.46)	10 (11.76)
Hyperlipidemia [n (%)]	16 (19.28)	9 (10.59)
Stroke [n (%)]	15 (18.07)	9 (10.59)
NYHA functional class		
Class I [n (%)]	0 (0.00)	1 (1.18)
Class II [n (%)]	50 (60.24)	58 (68.24)
Class III [n (%)]	25 (30.12)	19 (22.35)
Class IV [n (%)]	8 (9.64)	7 (8.24)
Type of heart failure		
HFrEF [n (%)]	9 (10.84)	7 (8.24)
HFmrEF [n (%)]	21 (25.30)	17 (20.00)
HFpEF [n (%)]	53 (63.86)	61 (71.76)
Type of atrial fibrillation		
Persistent AF [n (%)]	54 (65.06)	66 (77.65)
Paroxysmal AF [n (%)]	29 (34.94)	19 (22.35)

SGLT2i, Sodium-glucose cotransporter 2 inhibitors; AF, atrial fibrillation; HF, heart failure; NYHA functional class, The New York Heart Association (NYHA) Functional Classification; HFrEF, Heart Failure with reduced Ejection Fraction; HFmrEF, Heart Failure with Mid-Range Ejection Fraction; HFpEF, Heart failure with preserved ejection fraction.

**Table 2:** Comparison of laboratory indexes between the Two groups.

Laboratory parameter	SGLT2i group (*n* = 83)	Non-SGLT2i group (*n* = 85)
ALT[U/L,Mean ± SD]	29.08 ± 23.40	28.64 ± 21.11
AST[U/L,Mean ± SD]	26.17 ± 16.32	24.40 ± 11.54
Cr[*μ*mol/L,Mean ± SD]	81.60 ± 23.56	75.42 ± 16.69
eGFR[ml/min/1.73m2, median (IQR)]	93.69 (75.05, 116.22)	90.47 (75.61, 109.65)
LDL-C[mmol/L, median (IQR)]	2.67 (1.95, 3.31)	2.32 (1.77, 3.14)
hs-CRP[mg/L, median (IQR)]	3.40 (1.75, 10.51)	3.00 (2.40, 5.60)
Hb[g/L, Mean ± SD]	145.84 ± 17.21	141.35 ± 14.76
BNP[pg/mL, Mean ± SD]	312.23 ± 273.54	380.99 ± 364.52

SGLT2i, Sodium-glucose cotransporter 2 inhibitors.

**Table 3:** Comparison of operation-related indexes between the Two groups.

Procedure-related variable	SGLT2i group (*n* = 83)	Non-SGLT2i group (*n* = 85)
Degree of Left Atrial Fibrosis		
Severe Fibrosis [*n* (%)]	42 (50.60)	47 (55.29)
Mild Fibrosis [*n* (%)]	41 (48.24)	38 (44.71)
Ablation Surgical Strategy		
Circumferential Pulmonary Vein Isolation [*n* (%)]	83 (100.0)	85 (100.0)
Left Atrial Roof Line Ablation [*n* (%)]	48 (57.83)	54 (63.53)
Posterior Wall BOX Ablation [*n* (%)]	35 (42.17)	37 (43.53)
Tricuspid Isthmus Ablation [*n* (%)]	30 (36.14)	29 (34.12)
Mitral Isthmus Ablation [*n* (%)]	12 (14.46)	11 (12.94)

SGLT2i, Sodium-glucose cotransporter 2 inhibitors.

**Table 4:** Comparison of postoperative oral drugs between the Two groups.

Postoperative medication	SGLT2i group (*n* = 83)	Non-SGLT2i group (*n* = 85)
Anticoagulants [*n* (%)]	83 (100.0)	85 (100.0)
Antiarrhythmic drugs [*n* (%)]	73 (87.95)	74 (87.06)
Beta-blockers [*n* (%)]	50 (60.24)	39 (45.88)
ACEI/ARB/ARNI drugs [*n* (%)]	50 (60.24)	44 (51.76)
Aldosterone receptor antagonists [*n* (%)]	60 (72.29)	68 (80.00)

SGLT2i, Sodium-glucose cotransporter 2 inhibitors.

### 3.2 Atrial arrhythmia recurrence

#### 3.2.1 Atrial arrhythmia recurrence after catheter ablation

**Figure 1:**
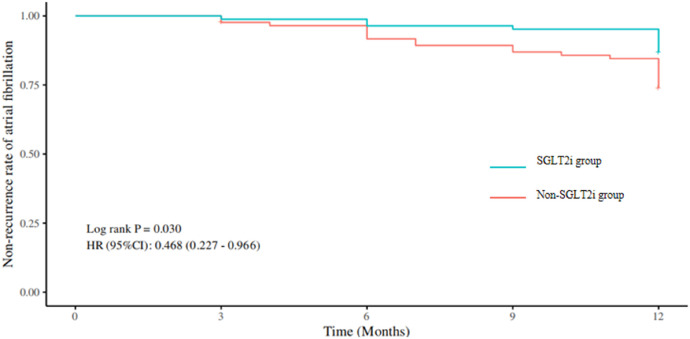
Kaplan–nnnnnMeier Curves of Non-Recurrence Rates of Postoperative Atrial Arrhythmias in the Two Groups. SGLT2i: Sodium-glucose cotransporter 2 inhibitors.

At 6 months post-catheter ablation: 3 out of 83 patients (3.61%) in the SGLT2i group experienced recurrence, compared to 7 out of 85 patients (8.24%) in the non-SGLT2i group. The atrial arrhythmia recurrence rate was slightly lower in the SGLT2i group, but the difference was not statistically significant (*P* = 0.347). At 12 months post-catheter ablation: 11 out of 83 patients (13.25%) in the SGLT2i group experienced recurrence, compared to 22 out of 85 patients (25.88%) in the non-SGLT2i group. The atrial arrhythmia recurrence rate was significantly lower in the SGLT2i group, and the difference was statistically significant (*P* = 0.039) (see Table 5 for details). Kaplan–Meier curves showed that the rate of freedom from atrial fibrillation recurrence was significantly higher in the SGLT2i group compared to the non-SGLT2i group, and the difference was statistically significant (HR 0.468, 95% CI: 0.227–0.966, *P* = 0.030). Please see Figure 1 for details.

**Table 5:** Comparison of postoperative recurrence of atrial arrhythmias between the Two groups.

Follow-up time	SGLT2i group	Non-SGLT2i group	*P* value
6 months[*n* (%)]	3 (3.61%)	7 (8.24)	0.347
12 months[*n* (%)]	11 (13.25%)	22 (25.88%)	0.039*

SGLT2i, Sodium-glucose cotransporter 2 inhibitors.

* Represents *P* < 0.05, statistically significant.

#### 3.2.2 Risk factor analysis for atrial arrhythmia recurrence after catheter ablation

Univariate Cox proportional hazards regression analysis showed that potential factors associated with atrial arrhythmia recurrence after catheter ablation included: persistent atrial fibrillation (HR 6.95, 95% CI: 1.66–29.05, *P* = 0.008), high-grade left atrial fibrosis (HR 4.16, 95% CI: 1.72–10.07, *P* = 0.002), left atrial (LA) size (HR 1.08, 95% CI: 1.01–1.15, *P* = 0.019); and SGLT2 inhibitor use (HR 0.47, 95% CI: 0.23–0.97, *P* = 0.040). Multivariate Cox proportional hazards regression analysis showed that high-grade left atrial fibrosis (HR 2.80, 95% CI: 1.13–6.96, *P* = 0.026) and SGLT2 inhibitor use (HR 0.45, 95% CI: 0.22–0.94, *P* = 0.033) were independent influencing factors for atrial arrhythmia recurrence after catheter ablation in patients with atrial fibrillation and heart failure. See Table 6 for details.

**Table 6:** Cox proportional hazards regression analysis of factors associated with postoperative recurrence of catheter ablation.

Risk factor	Univariate analysis			Multivariate analysis		
	HR	95%CI	P	HR	95%CI	P
Age	1.02	(0.99,1.06)	0.239	–	–	–
Male	0.67	(0.34,1.32)	0.244	–	–	–
BMI (kg/m2)	1.05	(0.96,1.16)	0.280	–	–	–
NYHA III-IV	0.81	(0.38,1.70)	0.574	–	–	–
HFrEF	1.02	(0.31,3.35)	0.969	–	–	–
Persistent AF	6.95	(1.66,29.05)	0.008*	3.96	(0.92,17.00)	0.064
Severe atrial fibrosis	4.16	(1.72,10.07)	0.002*	2.80	(1.13,6.96)	0.026*
hs-CRP (mg/L)	0.92	(0.84,1.00)	0.059	–	–	–
LAD (mm)	1.08	(1.01,1.15)	0.019*	1.05	(0.98,1.12)	0.188
LVEF (%)	1.00	(0.97,1.04)	0.867	–	–	–
SGLT2i	0.47	(0.23,0.97)	0.040*	0.45	(0.22,0.94)	0.033*

SGLT2i, Sodium-glucose cotransporter 2 inhibitors; LAD, Left atrial diameter; LVEF, Left Ventricular Ejection Fraction.

* Represents *P* < 0.05, statistically significant.

#### 3.2.3 Subgroup analysis for atrial arrhythmia recurrence after catheter ablation

**Figure 2:**
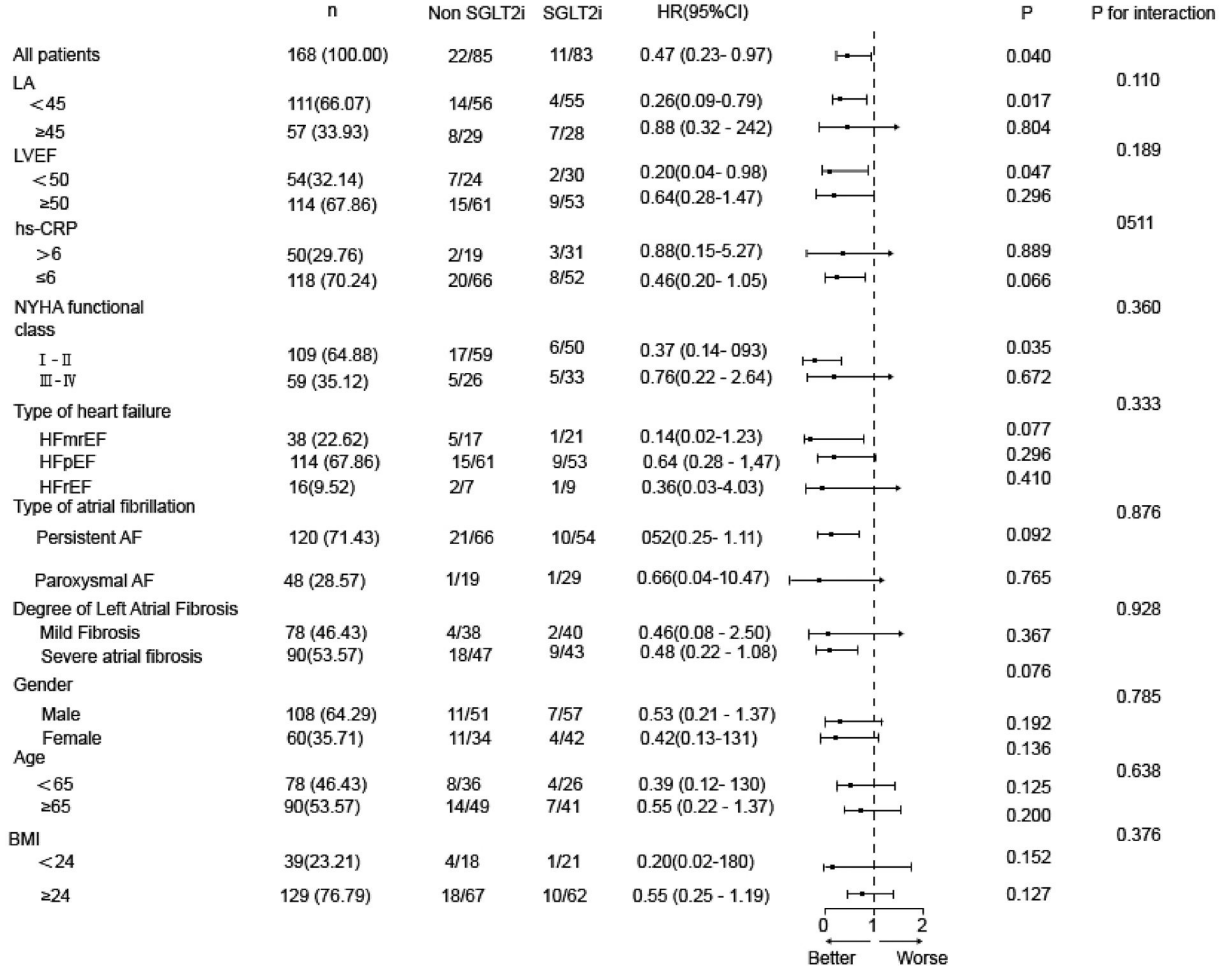
Forest plot for recurrence analysis of the atrial fibrillation subgroup.

Benefit analysis of SGLT2i was performed across subgroups defined by left atrial diameter, left ventricular ejection fraction, high-sensitivity C-reactive protein, NYHA functional class, heart failure type, atrial fibrillation type, left atrial fibrosis degree, gender, age, and BMI. We assessed atrial arrhythmia recurrence during follow-up in different subgroups. The results showed no significant interaction effects across subgroups (*P* > 0.05), indicating that SGLT2i could reduce atrial arrhythmia recurrence in all subgroup populations. Although interaction tests were not statistically significant (all *P* for interaction >0.05), point estimates suggested a numerically greater reduction in atrial arrhythmia recurrence among patients with LA <45 mm (HR 0.26, 95% CI: 0.09–0.79), LVEF <50% (HR 0.20, 95% CI: 0.04–0.98), and NYHA class I-II (HR 0.37, 95% CI: 0.14–0.93). These exploratory findings warrant confirmation in future prospective studies. See Figure 2 for details.

### 3.3 Comparison of cardiac function indicators between the Two groups

Baseline cardiac ultrasound indicators were largely consistent between the SGLT2i and non-SGLT2i groups, with no statistically significant differences (all *P* > 0.05). Comparisons of various echocardiographic parameters at 1, 3, and 6 months post-procedure showed no statistically significant differences between the two groups (all *P* > 0.05). At 12 months post-procedure, the SGLT2i group had a significantly smaller left atrial diameter (LA: 37.37 mm ± 4.25 mm vs. 39.25 mm ± 6.22 mm, *P* = 0.024) and a lower early diastolic mitral inflow velocity to mitral annular tissue velocity ratio (E/e': 10.71 ± 3.37 vs. 12.50 ± 5.50, *P* = 0.012) compared to the non-SGLT2i group. The left ventricular ejection fraction (LVEF: 62.06% ± 11.39% vs. 58.89% ± 7.98%, *P* = 0.039) was significantly higher in the SGLT2i group. All these differences were statistically significant. There was no significant improvement in left ventricular diameter (LV) or the ratio of early to late diastolic mitral inflow velocities (E/A ratio) compared to the non-SGLT2i group, and the differences were not statistically significant (*P* > 0.05). See Table 7 for details.

**Table 7:** Comparison of echocardiographic parameters during follow-up between SGLT2i and Non-SGLT2i groups.

Echocardiographic Parameter	Follow-up time	SGLT2i group (*n* = 83)	Non-SGLT2i group (*n* = 85)	P value
LAD[mm, Mean ± SD]	Preoperative	42.33 ± 5.39	41.86 ± 5.32	0.573
	1 month postoperative	39.89 ± 4.49	40.78 ± 7.07	0.306
	3 months postoperative	38.53 ± 5.50	39.78 ± 4.96	0.125
	6 months postoperative	38.07 ± 4.82	39.48 ± 5.01	0.065
	12 months postoperative	37.37 ± 4.25	39.25 ± 6.22	0.024*
LVD[mm, Mean ± SD]	Preoperative	50.48 ± 6.09	51.15 ± 6.83	0.503
	1 month postoperative	50.06 ± 4.51	50.48 ± 6.50	0.625
	3 months postoperative	49.33 ± 5.51	49.76 ± 8.13	0.683
	6 months postoperative	49.05 ± 5.06	49.59 ± 5.38	0.504
	12 months postoperative	48.76 ± 3.45	49.25 ± 5.69	0.501
LVEF[%, Mean ± SD]	Preoperative	52.26 ± 10.55	53.92 ± 10.06	0.299
	1 month postoperative	54.90 ± 9.26	56.97 ± 12.20	0.216
	3 months postoperative	58.86 ± 6.34	57.08 ± 10.44	0.182
	6 months postoperative	60.11 ± 5.47	58.12 ± 9.44	0.096
	12 months postoperative	62.06 ± 11.39	58.89 ± 7.98	0.039*
E/e’[Mean ± SD]	Preoperative	14.48 ± 7.66	15.42 ± 7.33	0.417
	1 month postoperative	13.80 ± 5.16	14.42 ± 5.82	0.472
	3 months postoperative	12.77 ± 4.74	13.37 ± 3.67	0.361
	6 months postoperative	11.61 ± 4.35	13.21 ± 6.19	0.053
	12 months postoperative	10.71 ± 3.37	12.50 ± 5.50	0.012*
E/A[Mean ± SD]	Preoperative	1.08 ± 0.33	1.12 ± 0.40	0.488
	1 month postoperative	1.11 ± 0.38	1.09 ± 0.47	0.829
	3 months postoperative	1.16 ± 0.41	1.14 ± 0.44	0.829
	6 months postoperative	1.25 ± 0.42	1.21 ± 0.47	0.548
	12 months postoperative	1.31 ± 0.39	1.27 ± 0.53	0.607

LAD, Left atrial diameter; LVD, Left ventricular diameter; LVEF, Left ventricular ejection fraction; E/e′, Ratio of early diastolic mitral inflow velocity to mitral annular tissue velocity; E/A, Ratio of early to late diastolic mitral inflow velocities; SGLT2i, Sodium-glucose cotransporter 2 inhibitors.

* Represents *P* < 0.05, statistically significant.

**Figure 3:**
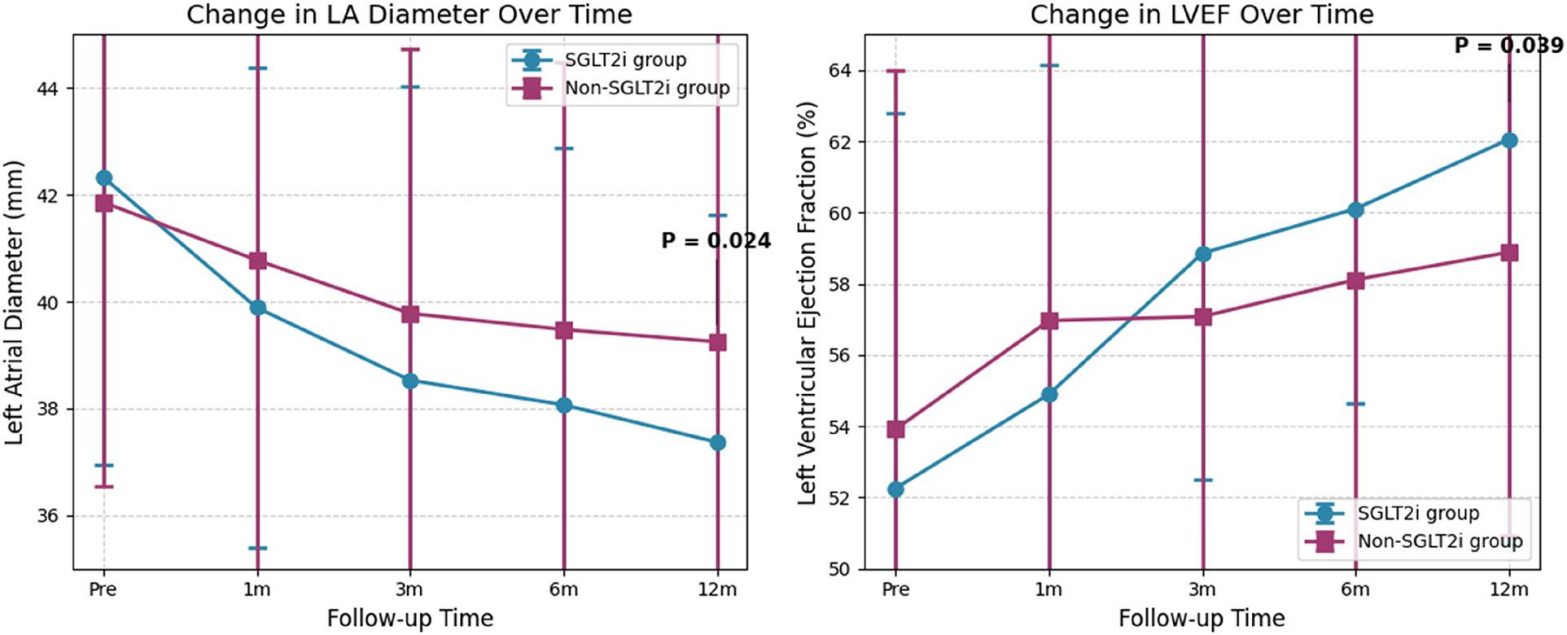
Line chart of echocardiographic indexes between the Two groups. Data are presented as mean ± SD. Left atrial diameter (LA) is shown in millimeters (mm); left ventricular ejection fraction (LVEF) is shown as percentage (%). The *Y*-axis for LA starts at 35 mm, and for LVEF starts at 50%, to reflect clinically meaningful changes. SGLT2i: Sodium-glucose cotransporter 2 inhibitors. *P < 0.05 for between-group comparison at 12 months. SGLT2i: Sodium-glucose cotransporter 2 inhibitors.

Repeated-measures analysis of variance showed a significant interaction between group and time for LA and LVEF. Simple effect analysis was performed on these, and line charts of cardiac ultrasound parameters at different time points were plotted. Compared to preoperative values, all cardiac ultrasound indicators in both groups showed an improving trend postoperatively. Compared to preoperative values, LA in the SGLT2i group was significantly reduced at 1, 3, 6, and 12 months postoperatively, and LVEF showed no significant increase at 1 month but was significantly increased at 3, 6, and 12 months postoperatively; these differences were statistically significant (*P* < 0.05). In the non-SGLT2i group, LA showed no significant reduction at 1 month but was significantly reduced at 3, 6, and 12 months postoperatively; LVEF showed no significant increase at 1 and 3 months but was significantly increased at 6 and 12 months postoperatively; these differences were statistically significant (*P* < 0.05). Compared to preoperative values, all echocardiographic parameters in both groups showed an improving trend. See Figure 3 for details.

## 4 Study part 2: meta-analysis materials and methods

The literature search, inclusion/exclusion criteria, data extraction, and statistical analysis for this study were conducted in accordance with the Preferred Reporting Items for Systematic Reviews and Meta-Analyses (PRISMA) statement (9). This study was not prospectively registered in PROSPERO because it was conceived as a hypothesis-generating analysis combining a single-center cohort with a literature review, rather than a standalone systematic review. Nevertheless, we adhered strictly to PRISMA guidelines to ensure transparency and reproducibility.

### 4.1 Inclusion and exclusion criteria

Inclusion Criteria (PICO):

1. Population: Patients > 18 years old with a clear diagnosis of atrial fibrillation (including paroxysmal or persistent AF) undergoing first-time catheter ablation, regardless of diabetes mellitus or heart failure status.
2. Intervention: Treatment with sodium-glucose cotransporter 2 (SGLT2) inhibitors. Experimental group: Received SGLT2 inhibitor treatment. Control group: Received non-SGLT2 inhibitor treatment.
3. Outcome Measures:
  • Primary outcome measures: Post-ablation atrial arrhythmia recurrence, atrial fibrillation (AF) recurrence.
  • Secondary outcome measures: Post-ablation all-cause mortality, hospitalization rate, and thromboembolic events.
4. Study Design: Randomized controlled trials (RCTs), cohort studies, or observational studies.

Studies meeting the inclusion criteria were required to have complete baseline data and provide data for the primary outcome measures.

Exclusion Criteria:

(1) Publications not in the English language.
(2) Commentaries, editorials, letters, reviews, conference abstracts, case reports, systematic reviews, and meta-analyses, or other types of literature.
(3) Studies for which the full text or original data could not be obtained.
(4) Original studies with duplicate data reporting and relatively less data; the study with more complete data was selected.
(5) Studies with incomplete baseline data or that did not report outcome measure data.
(6) Articles unrelated to the study content: This includes studies on SGLT2 inhibitor treatment in AF patients who did not undergo catheter ablation.

### 4.2 Search strategy

A literature search strategy was formulated based on the inclusion and exclusion criteria. PubMed, Embase, Cochrane Library, and Web of Science databases were systematically searched to identify relevant studies on SGLT2 inhibitors and post-ablation AF recurrence. The search covered the period from database inception to January 20, 2026. The language of included literature was restricted to English. Detailed searches of each database were performed using MeSH terms and relevant free-text keywords. Key search terms mainly included: [(atrial fibrillation) OR (AF) OR (AFib)] AND ((Sodium Glucose Transporter 2 Inhibitor) OR (SGLT2i) OR (Canagliflozin) OR (Dapagliflozin) OR (Empagliflozin) OR (Ipragliflozin) OR (Luseogliflozin) OR (Ertugliflozin) OR (Sotagliflozin) OR (Tofogliflozin) OR (Bexagliflozin)) AND [(Catheter Ablation) OR (Ablation)]. Detailed methodology is provided in Supplementary Table S1. Additionally, we manually reviewed the reference lists of all eligible studies to ensure the completeness of the search results.

### 4.3 Study selection and data extraction

Two researchers independently performed the literature search, selected studies for inclusion, extracted data, and cross-checked the extracted information. In cases of disagreement, a third researcher was consulted to reach a consensus. The following data were extracted from the included studies: first author, year of publication, country; study design, sample sizes for the control and experimental groups, type of AF, age, sex; study outcome measures, follow-up duration, etc. When duplicate publications of the same data existed, the original study with more complete data was selected for inclusion, and relevant information was extracted.

Outcome measures included: ① Differences in atrial arrhythmia recurrence and AF recurrence between the two groups during follow-up; ② Differences in atrial arrhythmia recurrence in subgroups of heart failure or diabetes; ③ Differences between the two groups in all-cause mortality, hospitalization rate, and thromboembolic events after ablation.

Atrial arrhythmia recurrence was defined as the occurrence of any episode of atrial fibrillation, atrial flutter, or atrial tachycardia lasting longer than 30 s, occurring after the 3-month blanking period following the ablation procedure, without concomitant use of antiarrhythmic drugs.

### 4.4 Literature quality assessment

The risk of bias in the included randomized controlled trials (RCTs) was assessed using the Cochrane Collaboration's Risk of Bias 2 (RoB 2) tool (10). The RoB 2 tool evaluates five domains: bias arising from the randomization process, bias due to deviations from intended interventions, bias due to missing outcome data, bias in measurement of the outcome, and bias in selection of the reported result. Each domain was judged as “low risk,” “some concerns,” or “high risk.” An overall risk-of-bias judgment was then derived for each RCT.

For the non-randomized studies (i.e., retrospective cohort studies), the Risk Of Bias In Non-randomized Studies of Interventions (ROBINS-I) tool was used (11). ROBINS-I assesses seven domains: confounding, selection of participants, classification of interventions, deviations from intended interventions, missing data, measurement of outcomes, and selection of the reported result. Each domain was rated as “low,” “moderate,” “serious,” or “critical” risk of bias, and an overall judgment was assigned.

Two reviewers independently performed the risk of bias assessments. Disagreements were resolved by discussion or consultation with a third reviewer.

### 4.5 Statistical analysis

Data processing and meta-analysis were performed using Stata 19 software. Heterogeneity across studies was examined using Cochran's *Q*-test and the *I*^2^ statistic. If the *P*-value for the *Q*-test was <0.10 or the *I*^2^ was >40%, significant heterogeneity was considered present. To reduce the impact of heterogeneity on the results, a random-effects model was applied to conduct all meta-analyses. Considering that the target outcomes were all dichotomous variables, the effect estimates were expressed as relative risks (RR) with their 95% confidence intervals (CI). Subgroup analyses were conducted based on patient population type (e.g., heart failure or type 2 diabetes) and different follow-up durations.

Sensitivity analysis was performed for the primary outcome measure using the leave-one-out method to verify the consistency of the conclusions when each study was removed in turn. If ten or more studies were included for an outcome, publication bias was assessed. Funnel plots were generated using STATA 19.0 statistical software, and Egger's test and Begg's test were performed. If the resulting *P*-values from both tests were ≥0.05, it suggested no significant publication bias. If the results of the two tests differed, the result of Egger's test was taken as the primary indicator.

## 5 Study part 2: meta-analysis results

### 5.1 Literature search results and basic characteristics of included studies

**Figure 4:**
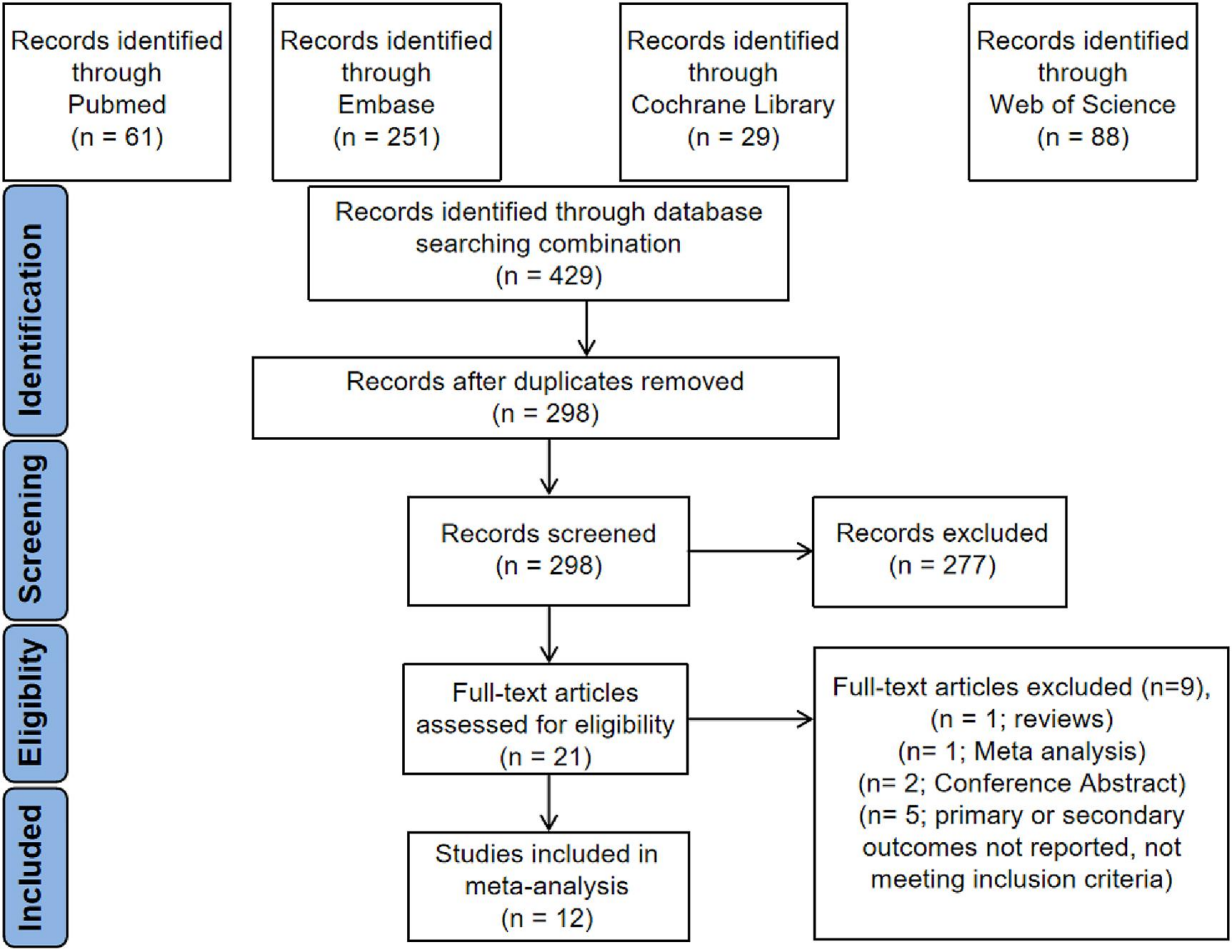
Flow chart of literature screening and results.

Initial searches identified 429 citations. After removing 131 duplicate records, 298 remained. Based on screening of titles and abstracts, 277 records were excluded, leaving 21 for full-text review. After reading the full texts, 9 studies were excluded (1 was a review, 1 was a meta-analysis, 2 were conference abstracts, and 5 did not meet inclusion criteria or did not report/extractable outcome data). Finally, 12 studies were included in the qualitative synthesis. The study selection process is illustrated in Figure 4. Among these, 2 were randomized controlled trials (RCTs) (12, 13) and 10 were retrospective cohort studies (RCS) (14–23). Two studies by Zhao et al. (14, 21) (covering distinct patient populations and time periods) and the study by Abu-Qaoud et al. (22) utilized propensity score matching in calculating outcome measures. The target population in the studies by Zhao et al. (14), Okajima et al. (15), Harada et al. (12), and Cetin et al. (18) consisted of individuals with AF and HF. In contrast, the studies by Qi et al. (19), Luo et al. (21), Zhao et al. (21), Abu-Qaoud et al. (22), and Kishima et al. (13) focused on AF individuals with T2DM. Both Harada et al. (12) and Qi et al. (19) restricted their analyses to individuals with persistent AF. Most included trials utilized both cryoballoon and radiofrequency energy sources for ablation. For paroxysmal AF, the predominant ablation strategy was bilateral pulmonary vein isolation. For persistent AF, in addition to PVI, additional linear ablation was performed as indicated. Individuals with concomitant atrial flutter also underwent cavotricuspid isthmus ablation. After incorporating our own cohort study, the meta-analysis ultimately included 13 studies (2 RCTs and 11 retrospective cohort studies), involving a total of 7,954 patients (SGLT2i group: 3,518; non-SGLT2i group: 4,436). A summary of the basic characteristics of the included studies is shown in Table 8.

**Table 8:** Basic Characteristics of the Included Studies.

Author	Publication Year	Study Design	Country	Diabetes Patients (%)	Heart Failure Patients (%)	Sample Size		Age		Male (%)		Persistent AF (%)		Follow-up (Months)
						SGLT2i group	Control group	SGLT2i group	Control group	SGLT2i group	Control group	SGLT2i group	Control group	
Gao	2026	RCS	China	44.12	100	83	85	63.73 ± 10.79	64.32 ± 8.99	57 (68.6)	51 (60.0)	54 (65.0)	66 (77.6)	12
Harada	2025	RCT	Japan	0	100	51	51	70.2 ± 8.3	72.0 ± 8.8	33 (64.8)	38 (74.5)	51 (100.0)	51 (100.0)	12
Kishima	2022	RCT	Japan	100	27.0	38	32	70.3 ± 8.6	70.3 ± 7.7	26 (68.0)	22 (69.0)	22 (58.0)	18 (56.0)	12
Zhao	2025	RCS (PSM)	China	51.63	100	368	368	63.5 ± 9.8	62.7 ± 10.9	242 (65.8)	239 (64.9)	279 (75.8)	291 (79.1)	27.5
Okajima	2025	RCS	Japan	15	100	45	96	NA	NA	NA	NA	NA	NA	12.4
Hakgor	2025	RCS	Turkey	24.1	18.4	211	403	59.8 ± 9.9	57.1 ± 9.7	129 (61.1)	286 (56.1)	129 (61.1)	286 (56.1)	24
Cetin	2025	RCS	Turkey	34.6	100	71	175	65.1 ± 7.9	63.1 ± 10.0	43 (60.6)	109 (62.3)	47.9	44.6	11.5
Qi	2024	RCS	China	100	8.8	91	91	70.9 ± 0.7	67.3 ± 0.9	52 (57.0)	48 (53.0)	91 (100.0)	91 (100.0)	16.2
Noh	2024	RCS	South Korea	31.5	NA	73	199	73.5 ± 4.8	71.7 ± 5.6	61 (83.6)	175 (87.9)	30 (41.1)	92 (46.2)	18
Luo	2024	RCS	China	100	NA	79	247	63.4 ± 10.4	63.8 ± 9.9	48 (60.8)	144 (58.3)	35 (44.3)	119 (48.2)	15.5
Zhao	2023	RCS (PSM)	China	100	35	138	387	63.9 ± 8.7	64.0 ± 9.5	98 (71.0)	276 (71.3)	64 (46.4)	203 (49.6)	15.5
Abu-Qaoud	2023	RCS (PSM)	United States	100	58.5	2,225	2,225	65.0 ± 9.0	65.0 ± 9.0	1,648 (75.0)	1,642 (74.0)	NA	NA	3∼12
Liu	2022	RCS	China	100	11	45	77	60.1 ± 10.6	63.2 ± 8.6	35 (78%)	54 (70%)	13 (29%)	23 (30%)	33.7 ± 21.6

SGLT2i, Sodium-glucose cotransporter 2 inhibitors; RCS, retrospective cohort studies; PSM, propensity score matching; RCT , randomized controlled trial; NA, not available.

### 5.2 Quality assessment

Randomized controlled trials: The two RCTs (Harada et al. and Kishima et al.) were assessed using the RoB 2 tool. Kishima et al. (13) showed low risk of bias in all domains, resulting in an overall low risk of bias. Harada et al. (12) had some concerns in the domain of bias due to deviations from intended interventions (lack of blinding of participants and personnel) and in the selection of the reported result, leading to an overall judgment of “some concerns.”

Non-randomized studies: The 11 retrospective cohort studies (including our own) were assessed using the ROBINS-I tool. As shown in Figure 8 and Supplementary Table S3, the majority of studies had moderate risk of bias, primarily due to potential confounding and selection of participants. Specifically, studies that did not use propensity score matching (e.g., Gao et al., Okajima et al., Hakgor et al.) were judged as having moderate risk of bias in the confounding domain. Studies that employed propensity score matching (Zhao 2023, Zhao 2025, Abu-Qaoud et al.) were rated as low to moderate risk. No study was judged as having serious or critical risk of bias. The overall ROBINS-I judgment for each study is summarized in Table 9.

**Table 9:** Risk of bias assessment for non-randomized studies using the ROBINS-I tool.

Study	year	Confounding	Selection of participants	Classification of interventions	Deviations from intended interventions	Missing data	Measurement of outcomes	Selection of reported result	Overall
Gao	2026	Moderate	Moderate	Low	Low	Low	Low	Low	Moderate
Zhao	2025	Low	Low	Low	Low	Low	Low	Low	Low
Okajima	2025	Moderate	Moderate	Low	Low	Low	Low	Low	Moderate
Hakgor	2025	Moderate	Moderate	Low	Low	Low	Low	Low	Moderate
Cetin	2025	Moderate	Low	Low	Low	Low	Low	Low	Moderate
Qi	2024	Moderate	Low	Low	Low	Low	Low	Low	Moderate
Noh	2024	Moderate	Low	Low	Low	Low	Low	Low	Moderate
Luo	2024	Moderate	Low	Low	Low	Low	Low	Low	Moderate
Zhao	2023	Low	Low	Low	Low	Low	Low	Low	Low
Abu-Qaoud	2023	Low	Low	Low	Low	Low	Low	Low	Low
Liu	2022	Moderate	Moderate	Low	Low	Low	Low	Low	Moderate

### 5.3 Efficacy outcomes

**Figure 5:**
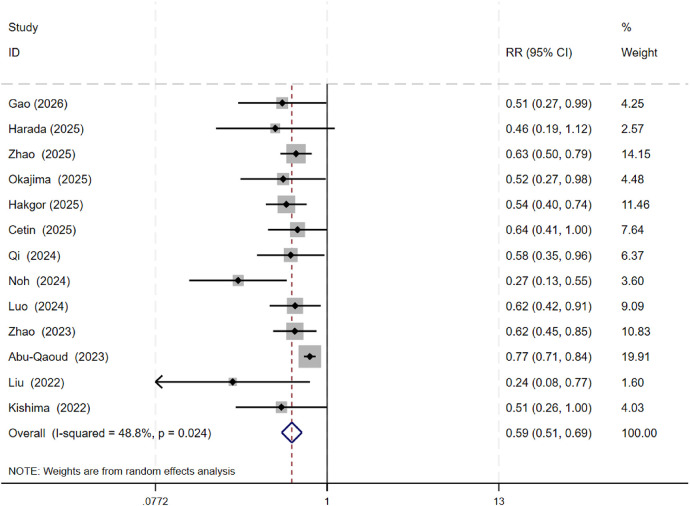
Meta-Analysis of atrial arrhythmia recurrence rates in the SGLT2i and Non-SGLT2i groups.

5.3.1. Atrial Arrhythmia Recurrence Risk in the Overall Population: Data from all 13 studies were analyzed. Among the 3,518 patients receiving SGLT2 inhibitor therapy who underwent catheter ablation, 879 experienced recurrence. Among the 4,436 control patients (not receiving SGLT2 inhibitor therapy after catheter ablation), 1,619 experienced recurrence. Heterogeneity testing indicated significant heterogeneity across studies (*P* = 0.024, *I*^2^ = 48.8%), therefore a random-effects model was used. Meta-analysis results indicated that in the overall population, the atrial arrhythmia recurrence rate after catheter ablation was significantly lower in the SGLT2 inhibitor group (24.99%) compared to the control group (36.50%), with the difference being statistically significant (RR = 0.59, 95% CI: 0.51 to 0.69, *P* < 0.001; see Figure 5). Sensitivity analysis using the leave-one-out method confirmed the stability of this result (see Supplementary Figure S1).

**Figure 6:**
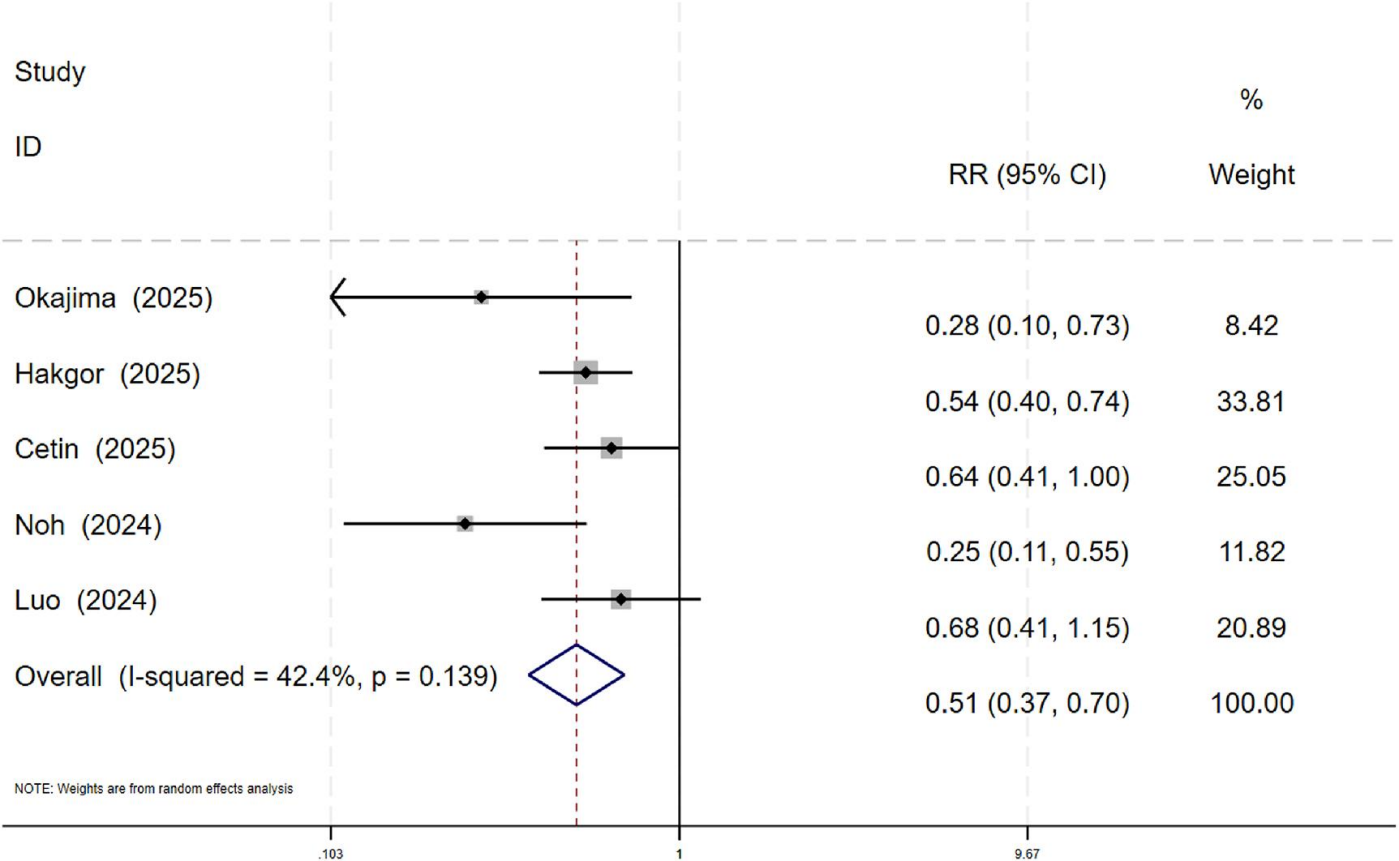
Meta-Analysis of atrial fibrillation recurrence rates in the SGLT2i and Non-SGLT2i groups.

5.3.2. Atrial Fibrillation Recurrence Risk: Five studies were included. Among the SGLT2 inhibitor group (479 patients), 83 experienced recurrence. Among the control group (1,120 patients), 374 experienced recurrence. Heterogeneity testing indicated significant heterogeneity across studies (*P* = 0.139, *I*^2^ = 42.4%), therefore a random-effects model was used. Meta-analysis results indicated that the atrial fibrillation recurrence rate after catheter ablation was significantly lower in the SGLT2 inhibitor group (17.33%) compared to the control group (33.39%), with the difference being statistically significant (RR = 0.51, 95% CI: 0.37 to 0.70, *P* < 0.001; see Figure 6).

### 5.4 Subgroup analyses

**Figure 7:**
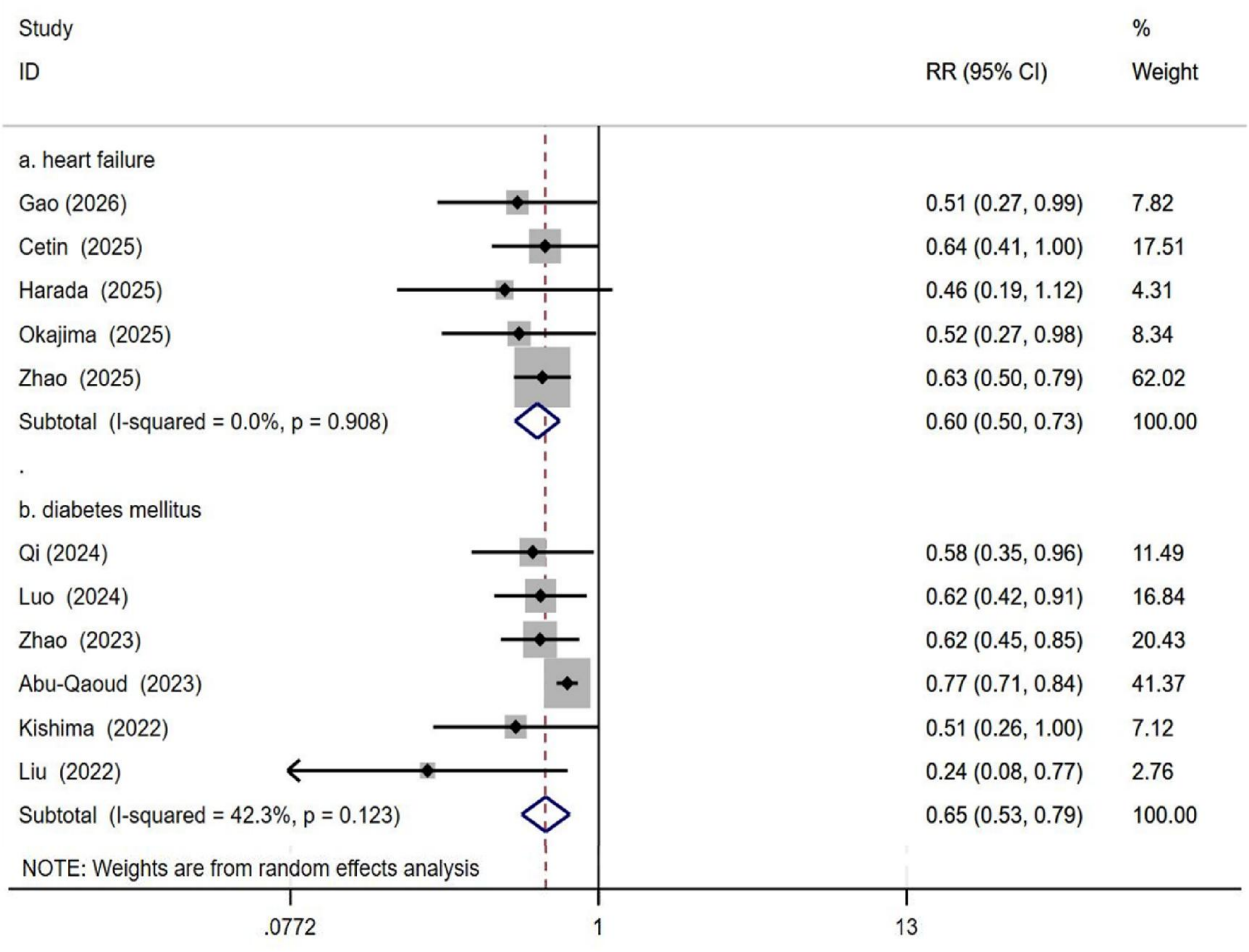
Impact of SGLT2i on AF recurrence after catheter ablation in patients with heart failure or diabetes Mellitus.

5.4.1. Based on Study Population: Analyses were divided into AF with HF and AF with T2DM groups. The AF with HF subgroup included 5 studies. The SGLT2i group comprised 618 patients, with 127 experiencing recurrence; the control group comprised 775 patients, with 273 experiencing recurrence. Using a random-effects model, SGLT2 inhibitors were associated with a reduced risk of post-ablation AF recurrence in HF patients (RR: 0.60; 95% CI: 0.50 to 0.73; *p* < 0.001; *I*^2^ = 0%; see Figure 7a). The AF with T2DM subgroup included 6 studies. Among 2,616 patients in the SGLT2i group, 704 experienced recurrence. Among 3,059 patients in the control group, 1,130 experienced recurrence. Using a random-effects model, SGLT2 inhibitors were associated with a reduced risk of post-ablation AF recurrence in diabetic patients (RR: 0.65; 95% CI: 0.53 to 0.79; *p* < 0.001; I² = 42.3%; see Figure 7b).

**Figure 8:**
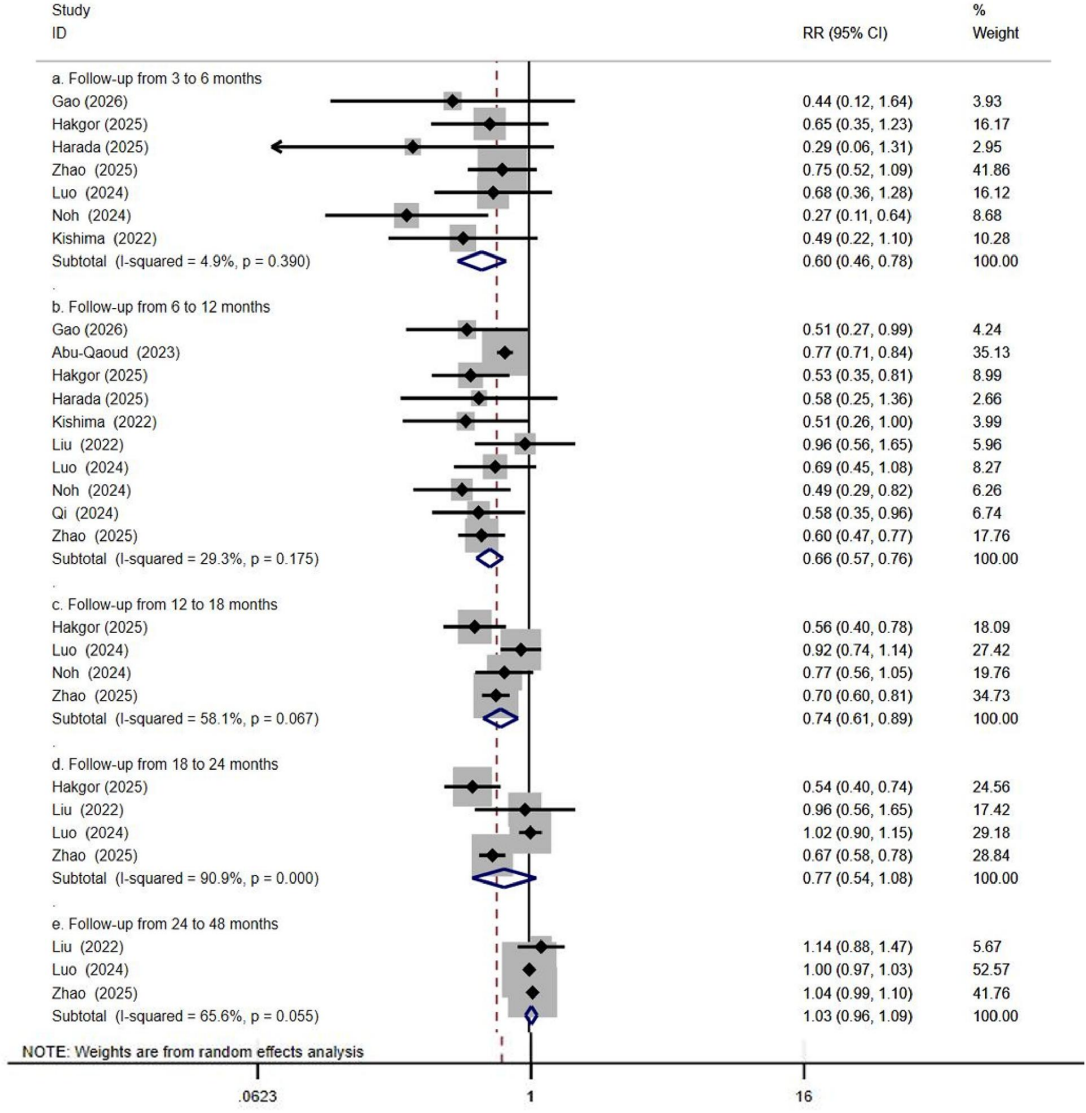
Impact of SGLT2i on AF recurrence after catheter ablation across different follow-up durations.

5.4.2. Based on Follow-up Duration: Groups were defined as: follow-up 3–6 months (Figure 8a), 6–12 months (Figure 8b), 12–18 months (Figure 8c), 18–24 months (Figure 8d), and 24–48 months (Figure 8e). Meta-analysis indicated that SGLT2 inhibitor treatment significantly reduced the risk of post-ablation atrial arrhythmia recurrence during follow-up periods of 3–6 months (RR: 0.60; 95% CI: 0.46 to 0.78; p* < 0.001; *I*^2^ = 4.9%), 6–12 months (RR: 0.60; 95% CI: 0.57 to 0.76; *p* < 0.001; *I*^2^ = 29.3%), and 12–18 months (RR: 0.74; 95% CI: 0.61 to 0.89; *p* = 0.001; *I*^2^ = 58.1%).

During follow-up periods of 18–24 months (RR: 0.77; 95% CI: 0.54 to 1.08; *P* = 0.132; *I*^2^ = 90.9%) and 24–48 months (RR: 1.03; 95% CI: 0.96 to 1.09; *P* = 0.422; *I*^2^ = 65.6%), the association between SGLT2i and reduced recurrence was not statistically significant. However, these null findings should be interpreted cautiously, as the limited number of studies with long-term follow-up may have resulted in type II error, and the high heterogeneity in the 18–24 month analysis further limits confidence in the pooled estimate.

### 5.5 Secondary outcome measures

**Figure 9:**
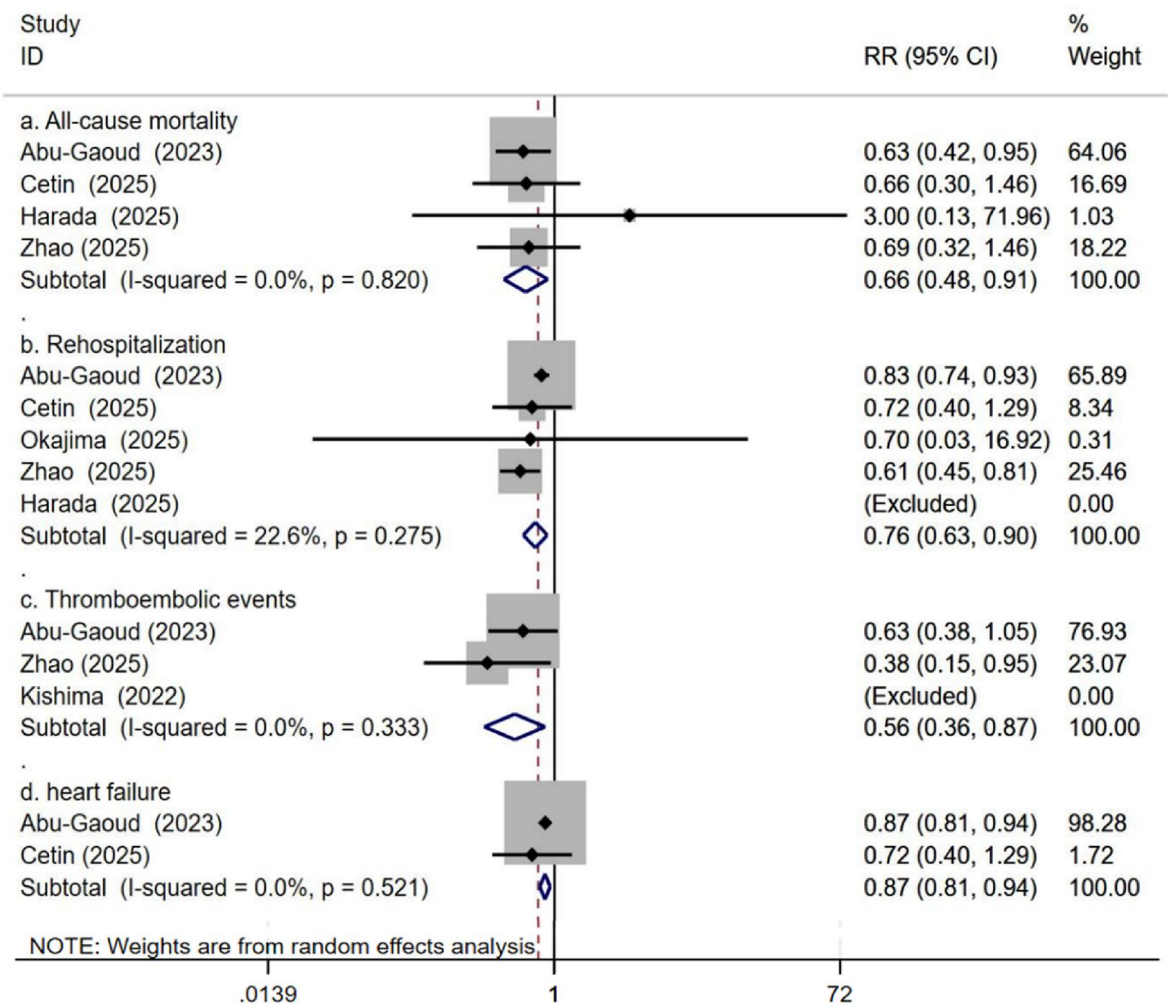
Impact of SGLT2i vs. Non-SGLT2i on All-Cause Mortality, Rehospitalization, Thromboembolic Events, and Heart Failure Events After Atrial Fibrillation Catheter Ablation.

5.5.1. All-Cause Mortality: Four studies analyzed the impact of SGLT2 inhibitors on all-cause mortality in AF patients after catheter ablation. Heterogeneity testing showed no significant heterogeneity across studies (*P* = 0.820, *I*^2^ = 0%). The results indicated that, compared to non-SGLT2i, SGLT2i use was associated with a reduction in all-cause mortality (RR: 0.66, 95% CI: 0.48 to 0.91, *P* = 0.011, see Figure 9a).

5.5.2. Rehospitalization: Five studies contributed data for this outcome. Heterogeneity across studies was low (*P* = 0.275, *I*^2^ = 22.6%). The results indicated that AF patients receiving SGLT2 inhibitor therapy had a lower incidence of rehospitalization after catheter ablation (RR: 0.76, 95% CI: 0.63 to 0.90, *P* = 0.002, see Figure 9b).

5.5.3. Thromboembolic Events: Three studies assessed this outcome. Heterogeneity testing showed no evidence of variability among studies (*P* = 0.333, I² = 0%). Consistently, individuals receiving SGLT2i experienced fewer thromboembolic events after catheter ablation (RR: 0.56, 95% CI: 0.36 to 0.87, *P* = 0.011, see Figure 9c).

5.5.4. Heart Failure-Related Events: Three studies assessed this outcome. Heterogeneity across studies was not significant (*P* = 0.521, *I*^2^ = 0%). The studies indicated that AF patients receiving SGLT2 inhibitor therapy had a lower incidence of heart failure-related events (HF worsening or HF rehospitalization) after catheter ablation (RR: 0.87, 95% CI: 0.81 to 0.94, *p* < 0.001, see Figure 9d).

### 5.6 Publication bias

For the primary outcome of atrial arrhythmia recurrence, which included 13 studies, visual inspection of the funnel plot suggested an asymmetrical distribution (see Supplementary Figure S2). Begg's test showed no statistically significant result (*t* = −1.89, *P* = 0.077). However, Egger's test (*t* = −4.27, *P* < 0.001) suggested potential publication bias. To further assess the impact of this bias on the primary outcome, the trim-and-fill method was applied under a random-effects model. The adjusted analysis using the trim-and-fill method suggested that publication bias may not have significantly altered the primary outcome (log RR: −0.595, 95% CI: −0.761 to −0.430) (see Supplementary Figure S3).

## 6 Discussion

In this study, the retrospective cohort analysis found that among patients with AF and HF undergoing first-time catheter ablation, treatment with SGLT2 inhibitors significantly reduced the risk of postoperative atrial arrhythmia recurrence. Multivariate Cox proportional hazards regression analysis identified left atrial fibrosis as an independent risk factor for atrial arrhythmia recurrence after catheter ablation in patients with AF and HF (HR 2.80, 95% CI: 1.13–6.96, *P* = 0.026). Conversely, SGLT2i use was an independent protective factor against atrial arrhythmia recurrence post-ablation in this population (HR 0.45, 95% CI: 0.22–0.94, *P* = 0.033). Subgroup analysis indicated that SGLT2i could reduce atrial arrhythmia recurrence across all subgroups. However, patients with LA <45 mm, LVEF <50%, NYHA class I-II HF, and severe atrial fibrosis appeared to derive benefit from SGLT2 inhibitor therapy after ablation. This represents a distinct highlight of our study compared to previously published research. But these findings should be interpreted with caution given the lack of significant interaction terms. Furthermore, another innovative aspect of our cohort study was the evaluation of cardiac function improvement at different follow-up time points (baseline, 3, 6, and 12 months post-procedure) in both the SGLT2i and non-SGLT2i groups. We found that in AF patients with HF post-catheter ablation, parameters such as LA diameter, E/e' ratio, and LVEF improved in both groups compared to baseline, with the degree of improvement increasing over time. The improvement was more pronounced in the SGLT2i group compared to the non-SGLT2i group. On the other hand, our meta-analysis, which is the most comprehensive to date in terms of included studies and data, further confirmed that early application of SGLT2i after catheter ablation significantly reduces the risk of atrial arrhythmia recurrence and improves prognosis in diverse AF populations, including those with HF.

With the aging global population, the incidence of atrial fibrillation is increasing worldwide. Statistics indicate that the global prevalence reached 50 million in 2020 (24, 25). By 2050, China is projected to have 9 million elderly AF patients over the age of 60 (26). AF increases the risk of death by 1.5–2 times and is associated with an elevated risk of multiple adverse outcomes. It increases the risk of stroke by 2.4 times, cognitive impairment or dementia by 1.5 times, myocardial infarction by 1.5 times, sudden cardiac death by 2 times, heart failure by 5 times, chronic kidney disease by 1.6 times, and peripheral arterial disease by 1.3 times (27–31). A study focusing on the elderly population showed that within 5 years of an AF diagnosis, there is an increased risk of new-onset heart failure (13.7%), new-onset stroke (7.1%), gastrointestinal bleeding (5.7%), and myocardial infarction (3.9%), among others (32). Therefore, how to reduce the occurrence and recurrence of AF remains a critical challenge to be explored and resolved.

Studies have found that more than half of HF patients develop AF, and more than one-third of AF patients develop HF (33). AF and HF frequently coexist. This is likely because they share common risk factors and comorbidities, including advanced age, hypertension, obesity, and coronary artery disease. Moreover, they share common triggering mechanisms, such as myocardial remodeling, activation of the renin-angiotensin-aldosterone system (RAAS) and sympathetic nervous system, inflammatory responses, and mitochondrial dysfunction. These factors can promote the simultaneous occurrence of both conditions. Furthermore, they interact, forming a vicious cycle. During AF, the loss of effective atrial contraction and the rapid, irregular ventricular rate can induce and accelerate HF progression. In HF, elevated left atrial pressure promotes atrial fibrosis, leading to atrial structural and electrical remodeling, which further induces and maintains AF (34). AF and HF can mutually promote each other, creating a cause-and-effect relationship that poses significant challenges for clinical management.

Catheter ablation has become a first-line treatment for atrial fibrillation (35, 36). For AF patients with concomitant HF, maintaining sinus rhythm through catheter ablation can thereby improve left ventricular function (37). While radiofrequency and cryoballoon ablation remain the standard of care, emerging technologies such as pulsed-field ablation (PFA) offer potential advantages in procedural safety and efficiency, although their impact on long-term recurrence rates in HF patients requires further investigation. AF is typically initiated by ectopic foci originating from the pulmonary veins in the left atrium (38). Therefore, strategies such as point-by-point radiofrequency or cryoballoon ablation or pulsed-field ablation to achieve pulmonary vein isolation (PVI) form the cornerstone of AF ablation. However, for persistent AF, due to the frequent presence of extra-pulmonary vein triggers, PVI alone is often insufficient, and additional linear ablation strategies are commonly combined in clinical practice. Studies show that the single-procedure success rate for persistent AF ablation is 43%, while multiple procedures or combination with antiarrhythmic drugs can increase the success rate to 69% (31). Post-ablation recurrence rates remain high, making optimal post-ablation pharmacological management crucial for reducing AF recurrence and increasing postoperative sinus rhythm maintenance rates.

Sodium-glucose cotransporter 2 inhibitors (SGLT2i) work by inhibiting SGLT2 in the renal proximal tubules, reducing the reabsorption of glucose and sodium, increasing urinary glucose excretion, and thereby effectively lowering blood glucose levels (39). Research has also uncovered cardiorenal benefits of SGLT2i independent of their glucose-lowering effects. SGLT2i can effectively reduce serum creatinine and uric acid, slow the progression of renal failure, and lower renal disease mortality (40, 41). They are recommended as first-line therapy for chronic kidney disease in multiple guidelines, regardless of T2DM status (42). In cardiovascular therapy, SGLT2i can reduce acute myocardial infarction, lower the risk of heart failure hospitalization and cardiovascular death (43, 44), and have been recommended as Class IA therapy for heart failure across the full ejection fraction spectrum in several guidelines. Recent studies have found that SGLT2i possess potential in treating arrhythmias, particularly in preventing and managing AF (45). SGLT2i may modulate the development and progression of AF through mechanisms such as: regulating Na⁺ and Ca^2+^ disturbances in atrial cardiomyocytes, improving mitochondrial function, suppressing inflammatory responses, reducing atrial myocardial fat infiltration, modulating neurohormonal pathways, providing energy supply, reducing cardiac load, and optimizing hemodynamic status (46). A substantial body of animal experiments and clinical research exploration shows that SGLT2i can reduce AF occurrence and inhibit its progression. In inducing AF in mouse or canine models, empagliflozin, canagliflozin, and dapagliflozin have all demonstrated inhibitory effects on AF initiation and development (5, 6, 47). A secondary analysis of the DECLARE-TIMI 58 trial found that T2DM patients treated with dapagliflozin had a 19% lower risk of new-onset atrial fibrillation/atrial flutter (AF/AFL) (264 vs. 325; HR 0.81, 95% CI: 0.68–0.95, *P* = 0.009) (45). A meta-analysis indicated that SGLT2 inhibitors reduced the risk of AF/AFL occurrence in T2DM patients by 24% (RR 0.76, 95% CI: 0.65–0.90, *P* = 0.001) (48). A study from Kaohsiung Medical University Hospital showed that among hypertensive T2DM patients, those receiving SGLT2 inhibitor therapy had significantly lower total arrhythmia incidence (HR 0.58, 95% CI: 0.38–0.89, *P* = 0.013) and AF incidence (HR 0.56, 95% CI: 0.35–0.88, *P* = 0.013) (49).

To further investigate whether AF patients with HF benefit from taking SGLT2i after catheter ablation, we conducted the relevant study. After 12 months of follow-up, the atrial arrhythmia recurrence rate was significantly lower in the SGLT2i group compared to the non-SGLT2i group (13.25% vs. 25.88%, *P* = 0.039). Our study suggests that SGLT2i can significantly reduce the risk of AF recurrence after ablation in AF patients with HF. Univariate COX regression analysis showed that SGLT2i use was associated with a reduced risk of AF recurrence (HR 0.47, 95% CI: 0.23–0.97, *P* = 0.040). After adjusting for confounders such as persistent AF, high-grade left atrial fibrosis, and left atrial size in multivariate COX regression analysis, SGLT2i still significantly reduced the AF recurrence rate (HR 0.45, 95% CI: 0.22–0.94, *P* = 0.033). These results are largely consistent with previous studies. Additionally, prior research found that the beneficial effect of SGLT2i on AF recurrence was particularly evident in male patients and those with persistent AF (50). Neither the subgroup analysis in our cohort study nor in the meta-analysis found differential effects of gender, AF type, or the presence of HF or diabetes on the ability of SGLT2i to reduce postoperative AF recurrence. This may be related to the relatively small sample size in the cohort study and the lack of granular subdivision in the meta-analysis subgroups. Our exploratory subgroup analysis suggested that patients with LA < 45 mm, LVEF < 50%, and NYHA class I-II may derive a more pronounced association with reduced recurrence, but these findings should be interpreted with caution given the lack of significant interaction terms. This cohort analysis suggested that AF patients with a high degree of atrial fibrosis might benefit from SGLT2i, but no statistically significant difference was observed, possibly due to our lack of more detailed stratification of fibrosis degree. This may indicate that the efficacy of SGLT2i in improving AF recurrence exhibits individual variability among AF patients with different degrees of atrial fibrosis.

Regarding the improvement of cardiac function and prognosis, the study by John et al. showed that dapagliflozin effectively improved cardiovascular adverse events in HF patients (HR 0.74, 95% CI: 0.65–0.85, *P* < 0.001), and results were similar for diabetic and non-diabetic patients (51). Packer et al. found that the beneficial effect of empagliflozin in reducing cardiovascular death or hospitalization for heart failure was largely similar to the above findings, regardless of diabetes status (HR 0.75, 95% CI: 0.65–0.86, *P* < 0.001) (52). Our meta-analysis further supports these findings, indicating that SGLT2i drugs can reduce the risk of heart failure worsening or death from cardiovascular causes, as well as the risk of rehospitalization, in AF patients with HF after catheter ablation.

Although previous studies have confirmed the definitive therapeutic effect of SGLT2i on HF and their ability to reduce cardiovascular adverse events such as HF rehospitalization and mortality, it remains unclear whether SGLT2i application after catheter ablation can improve cardiac function specifically in AF patients with HF undergoing first-time ablation. Soga et al. found that T2DM patients with stable HF showed significant improvement in cardiac function indices, including E/e' and LVEF, after 6 months of dapagliflozin treatment (53). Our study is the first to record and analyze echocardiographic parameters at 1, 3, 6, and 12 months post-catheter ablation, further investigating the impact of SGLT2i on cardiac function in AF patients with HF after ablation. Unlike the study by Soga et al., at 6 months post-procedure in our study, the SGLT2i group showed a decrease in E/e′ and an increase in LVEF, but the differences compared to the non-SGLT2i group were not statistically significant. However, at 12 months post-procedure, compared to the non-SGLT2i group, the SGLT2i group had a significantly smaller LA diameter (37.37 ± 4.25 mm vs. 39.25 ± 6.22 mm, *P* = 0.024), a significantly lower E/e' ratio (10.71 ± 3.37 vs. 12.50 ± 5.50, *P* = 0.012), and a significantly higher LVEF (62.06 ± 11.39% vs. 58.89 ± 7.98%, *P* = 0.039). Since no other studies have systematically evaluated the effect of SGLT2i on cardiac function at different time points, a meta-analysis on this specific aspect was not performed.

Our findings suggest that for AF patients post-catheter ablation, SGLT2i may have a positive effect in reducing atrial fibrillation recurrence and improving cardiac function. SGLT2i should be initiated as early as possible. However, its beneficial effect on improving cardiac function in HF patients takes a relatively long time to manifest, therefore long-term application of SGLT2i is recommended. Nevertheless, the meta-analysis found that during the 24–48 month follow-up period post-ablation, the risk of AF recurrence in patients using SGLT2 inhibitors was similar to those not using them. This may be related to reasons such as the slow progression of atrial fibrosis over time with prolonged AF duration, progressive decline in cardiac function, or increasing comorbidities. However, our cohort study did not have such a long follow-up period. Therefore, whether the efficacy of SGLT2i in reducing AF recurrence and improving cardiac function is time-limited requires long-term follow-up in multicenter randomized controlled trials. Furthermore, analysis is needed to determine whether there are differential effectiveness of SGLT2i in AF patients with varying degrees of atrial fibrosis, to further strengthen individualized treatment and maximize benefits for AF patients.

## 7 Conclusion

We conducted two studies: a retrospective cohort analysis and a meta-analysis. The results from both studies consistently suggest a positive association between SGLT2 inhibitors and reduced atrial arrhythmia recurrence, as well as improved cardiac function, in AF patients with HF after catheter ablation. SGLT2i is also associated with lower risks of all-cause mortality, rehospitalization, and HF events. However, considering the limitations of the current research, future prospective studies, including multicenter randomized controlled trials, are needed. These should extend follow-up durations and include diverse patient populations to validate the individualized efficacy and variability of SGLT2i in atrial fibrillation patients undergoing catheter ablation.

## 8 Limitations and shortcomings

1. The cohort analysis in this study was a single-center, retrospective investigation with a relatively small sample size, which may introduce bias into the results. Furthermore, the follow-up period was limited to 12 months, precluding assessment of the very long-term effectiveness of SGLT2i.
2. Compared to long-term continuous monitoring, the use of routine bedside electrocardiography and 24-hour Holter monitoring may underestimate the rate of atrial arrhythmia recurrence.
3. This study did not analyze adverse cardiovascular outcomes such as rehospitalization rates or cardiovascular mortality in atrial fibrillation patients with heart failure.
4. Patients in this study were administered two different types of SGLT2i, dapagliflozin and empagliflozin. Potential differences in the effects of different drug types may have influenced the experimental results.
5. No detailed analysis of atrial fibrosis was performed in the atrial fibrillation patients to evaluate the differential impact of SGLT2i on postoperative recurrence in patients with varying degrees of atrial fibrosis.
6. Future research should involve larger sample sizes and conduct multicenter, large-scale prospective studies focusing on a single drug. These studies should extend follow-up periods, closely monitor patient electrocardiographic changes, and tabulate cardiovascular outcome events to further investigate the beneficial effects of different drugs in atrial fibrillation patients with diverse characteristics.
7. Due to the retrospective design and limited sample size, propensity score matching was not feasible. The absence of propensity score matching in the cohort study may not fully eliminate treatment selection bias. Although multivariable adjustment was applied, residual confounding by unmeasured factors (e.g., disease severity, physician preference) cannot be excluded.
8. The combined analysis of dapagliflozin and empagliflozin assumes class effects; potential differences between individual SGLT2i drugs could not be evaluated.

## Data Availability

The original contributions presented in the study are included in the article/Supplementary Material, further inquiries can be directed to the corresponding author.
