# Supplementary material for: The effects of SGLT2 inhibitors on atrial fibrillation recurrence and cardiac function after catheter ablation in patients with atrial fibrillation and heart failure: a retrospective cohort study and meta-analysis
Source: Front Cardiovasc Med. 2026 May 1;13:1827339. doi: 10.3389/fcvm.2026.1827339 (PMC13175794; doi:10.3389/fcvm.2026.1827339)

Supplementary material:

Table 1: Detailed search terms in different databases

Database	Retrieval Strategies	Result
1.PUBMED	#1 (Atrial Fibrillation[mh]) OR (AF) OR (Atrial Fibrillations) OR (Fibrillation, Atrial) OR (Fibrillations, Atrial) OR (Auricular Fibrillation) OR (Auricular Fibrillations) OR (Fibrillation, Auricular) OR (Fibrillations, Auricular) OR (Persistent Atrial Fibrillation) OR (Atrial Fibrillation, Persistent) OR (Atrial Fibrillations, Persistent) OR (Fibrillation, Persistent Atrial) OR (Fibrillations, Persistent Atrial) OR (Persistent Atrial Fibrillations) OR (Familial Atrial Fibrillation) OR (Atrial Fibrillation, Familial) OR (Atrial Fibrillations, Familial) OR (Familial Atrial Fibrillations) OR (Fibrillation, Familial Atrial) OR (Fibrillations, Familial Atrial) OR (Paroxysmal Atrial Fibrillation) OR (Atrial Fibrillation, Paroxysmal) OR (Atrial Fibrillations, Paroxysmal) OR (Fibrillation, Paroxysmal Atrial) OR (Fibrillations, Paroxysmal Atrial) OR (Paroxysmal Atrial Fibrillations) 26,007<br>#2 (Sodium Glucose Transporter 2 Inhibitors[mh]) OR (Sodium – Glucose Cotransporter 2 Inhibitor) OR (SGLT-2 Inhibitors OR SGLT 2 Inhibitors) OR (SGLT-2 Inhibitor) OR (Inhibitor,SGLT-2) OR (SGLT 2 Inhibitor) OR (Sodium-Glucose Transporter 2 Inhibitor) OR (Sodium Glucose Transporter 2 Inhibitor) OR (SGLT2 Inhibitor) OR (Inhibitor, SGLT2) OR (Gliflozins) OR (Gliflozin) OR (SGLT2 Inhibitors) OR (SGLT2i) OR (Canagliflozin) OR (Dapagliflozin) OR (Empagliflozin) OR (Ipragliflozin) OR (Luseogliflozin) OR (Ertugliflozin) OR (Ertugliflozin) OR (Sotagliflozin) OR (Tofogliflozin) OR (Bexagliflozin) 6,160<br>#3 (Catheter Ablation) OR (Ablation) 22,275<br>#4 #1 AND #2 AND #3 61	61
2.EMBASE	#1 'atrial fibrillation':ti,ab,kw OR af:ti,ab,kw OR 'auricular fibrillation':ti,ab,kw OR afib:ti,ab,kw 245,883<br>#2 'Atrial Fibrillation'/exp 271,013<br>#3 'sodium glucose transporter 2 inhibitors':ti,ab,kw OR sglt2i:ti,ab,kw OR canagliflozin:ti,ab,kw OR dapagliflozin: ti,ab,kw OR empagliflozin:ti,ab,kw OR ipragliflozin:ti,ab,kw OR luseogliflozin:ti,ab,kw OR ertugliflozin:ti,ab,kw OR sotagliflozin:ti,ab,kw OR tofogliflozin:ti,ab,kw OR bexagliflozin:ti,ab,kw 23,389<br>#4 'Sodium Glucose Transporter 2 Inhibitors'/exp 45,094	251

	#5 'catheter ablation':ti,ab,kw OR ablation:ti,ab,kw 222,756<br>#6 'Catheter Ablation'/exp 51,919<br>#7 #1 OR #2 324,066<br>#8 #3 OR #4 46,657<br>#9 #5 OR #6 230,967<br>#10 #8 AND #9 AND #10 251	
3.The Cochrane Library	#1 (Atrial Fibrillation) OR (AF) OR (Atrial Fibrillations) OR (Fibrillation, Atrial) OR (Fibrillations, Atrial) OR (Auricular Fibrillation) OR (Auricular Fibrillations) OR (Fibrillation, Auricular) OR (Fibrillations, Auricular) OR (Persistent Atrial Fibrillation) OR (Atrial Fibrillation, Persistent) OR (Atrial Fibrillations, Persistent) OR (Fibrillation, Persistent Atrial) OR (Fibrillations, Persistent Atrial) OR (Persistent Atrial Fibrillations) OR (Familial Atrial Fibrillation) OR (Atrial Fibrillation, Familial) OR (Atrial Fibrillations, Familial) OR (Familial Atrial Fibrillations) OR (Fibrillation, Familial Atrial) OR (Fibrillations, Familial Atrial) OR (Paroxysmal Atrial Fibrillation) OR (Atrial Fibrillation, Paroxysmal) OR (Atrial Fibrillations, Paroxysmal) OR (Fibrillation, Paroxysmal Atrial) OR (Fibrillations, Paroxysmal Atrial) OR (Paroxysmal Atrial Fibrillations) 20,590<br>#2 MeSH descriptor: [Atrial Fibrillation] explode all trees 7,638<br>#3 (Sodium Glucose Transporter 2 Inhibitors) OR (Sodium－Glucose Cotransporter 2 Inhibitor) OR (SGLT-2 Inhibitors OR SGLT 2 Inhibitors) OR (SGLT-2 Inhibitor) OR (Inhibitor,SGLT-2) OR (SGLT 2 Inhibitor) OR (Sodium-Glucose Transporter 2 Inhibitor) OR (Sodium Glucose Transporter 2 Inhibitor) OR (SGLT2 Inhibitor) OR (Inhibitor, SGLT2) OR (Gliflozins) OR (Gliflozin) OR (SGLT2 Inhibitors) OR (SGLT2i) OR (Canagliflozin) OR (Dapagliflozin) OR (Empagliflozin) OR (Ipragliflozin) OR (Luseogliflozin) OR (Ertugliflozin) OR (Ertugliflozin) OR (Sotagliflozin) OR (Tofogliflozin) OR (Bexagliflozin) 7,586<br>#4 MeSH descriptor: [Sodium-Glucose Transporter 2 Inhibitors] explode all trees 1,271<br>#5 (Catheter Ablation) OR (Ablation) 14,206<br>#6 MeSH descriptor: [Catheter Ablation] explode all trees 0<br>#7 (#1 OR #2) AND (#3 OR #4) AND (#5 OR #6) 29	29
4.SCI-Web of Science	#1 TS=((Atrial Fibrillation) OR (AF) OR (Atrial Fibrillations) OR (Fibrillation, Atrial) OR (Fibrillations, Atrial) OR (Auricular Fibrillation) OR (Auricular Fibrillations) OR (Fibrillation, Auricular) OR (Fibrillations, Auricular) OR	88

	(Persistent Atrial Fibrillation) OR (Atrial Fibrillation, Persistent) OR (Atrial Fibrillations, Persistent) OR (Fibrillation, Persistent Atrial) OR (Fibrillations, Persistent Atrial) OR (Persistent Atrial Fibrillations) OR (Familial Atrial Fibrillation) OR (Atrial Fibrillation, Familial) OR (Atrial Fibrillations, Familial) OR (Familial Atrial Fibrillations) OR (Fibrillation, Familial Atrial) OR (Fibrillations, Familial Atrial) OR (Paroxysmal Atrial Fibrillation) OR (Atrial Fibrillation, Paroxysmal) OR (Atrial Fibrillations, Paroxysmal) OR (Fibrillation, Paroxysmal Atrial) OR (Fibrillations, Paroxysmal Atrial) OR (Paroxysmal Atrial Fibrillations)) 200,674<br>#2 TS=((Sodium Glucose Transporter 2 Inhibitors) OR (Sodium ⁻ Glucose Cotransporter 2 Inhibitor) OR (SGLT-2 Inhibitors OR SGLT 2 Inhibitors) OR (SGLT-2 Inhibitor) OR (Inhibitor,SGLT-2) OR (SGLT 2 Inhibitor) OR (Sodium-Glucose Transporter 2 Inhibitor) OR (Sodium Glucose Transporter 2 Inhibitor) OR (SGLT2 Inhibitor) OR (Inhibitor, SGLT2) OR (Gliflozins) OR (Gliflozin) OR (SGLT2 Inhibitors) OR (SGLT2i) OR (Canagliflozin) OR (Dapagliflozin) OR (Empagliflozin) OR (Ipragliflozin) OR (Luseogliflozin) OR (Ertugliflozin) OR (Ertugliflozin) OR (Sotagliflozin) OR (Tofogliflozin) OR (Bexagliflozin)) 25,940<br>#3 TS=((Catheter Ablation) OR (Ablation)) 268,156<br>#4 #1 AND #2 AND #3 88	

Supplementary material: Figure 1 Sensitivity analysis of meta-analysis on recurrence of atrial arrhythmia

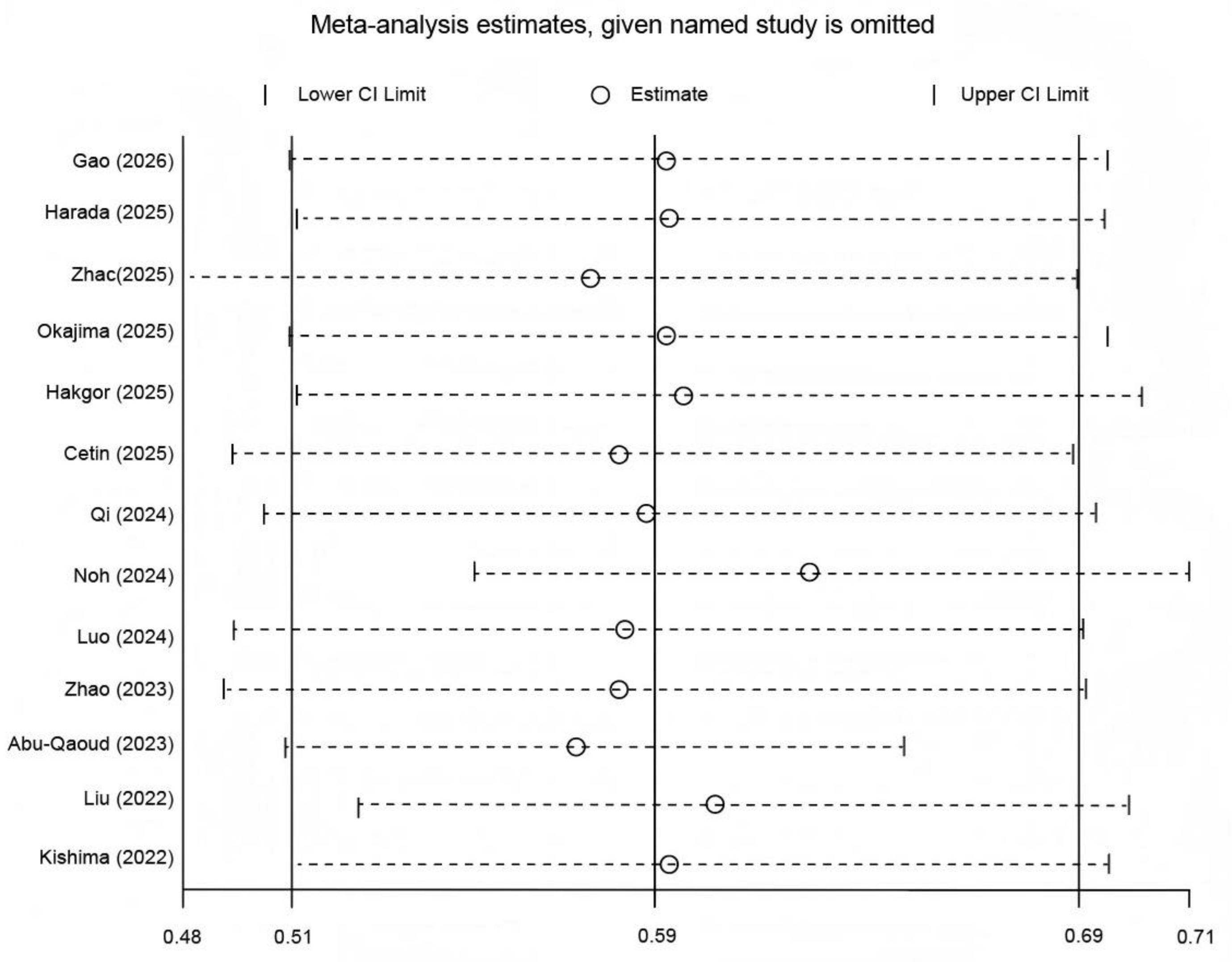

Supplementary material: Figure 2 Funnel plot assessing publication bias for atrial arrhythmia recurrence.

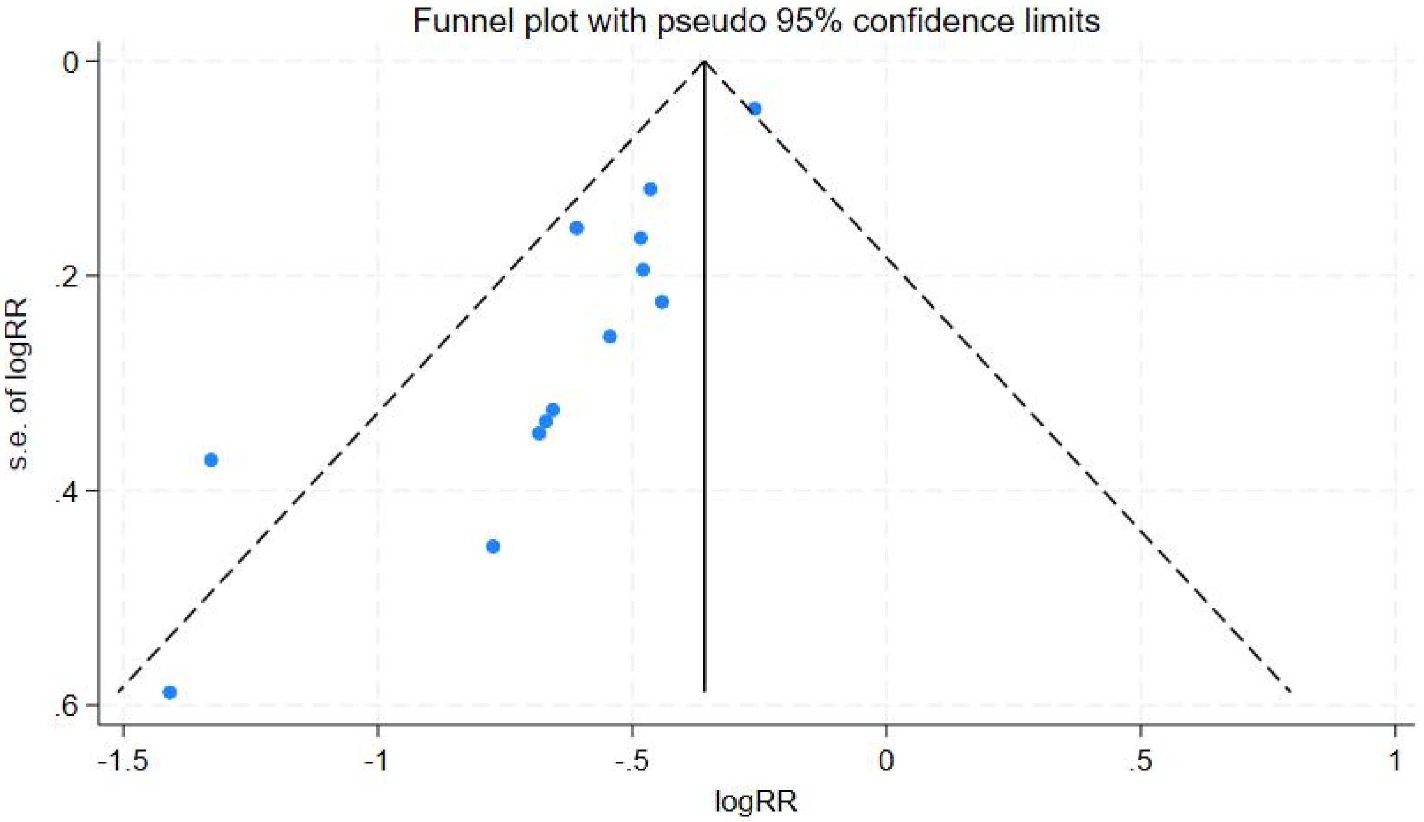

Supplementary material: Figure 3 Trim-and-fill analysis evaluating the influence of publication bias on atrial arrhythmia

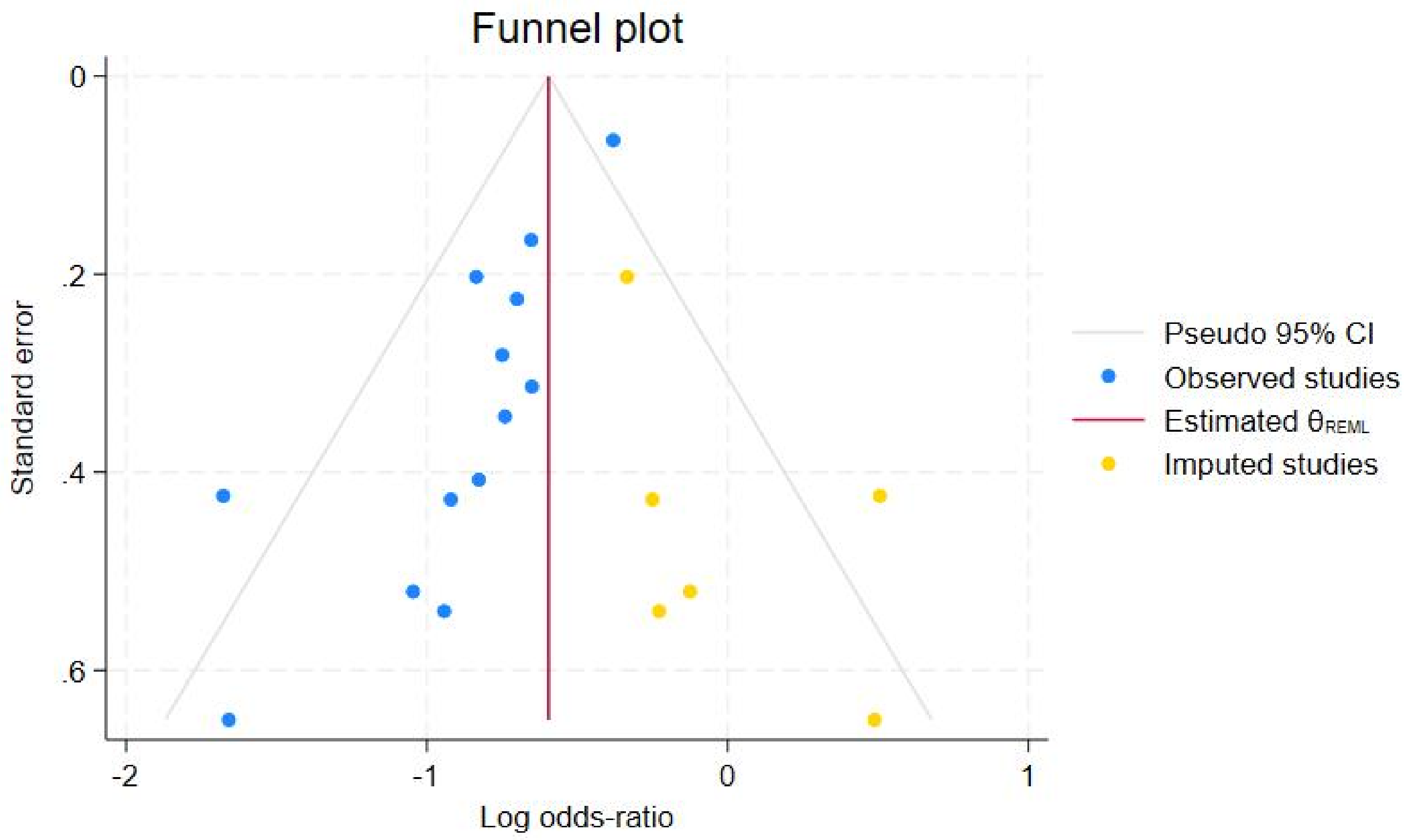

Supplement: Supplementary file 1 [file Datasheet1.pdf]
